# Neuroinflammation causes mitral cell dysfunction and olfactory impairment in a multiple sclerosis model

**DOI:** 10.1186/s12974-025-03388-5

**Published:** 2025-03-08

**Authors:** Charlotte Schubert, Kristina Schulz, Jana K. Sonner, Alexandros Hadjilaou, Anna-Lena Seemann, Janine Gierke, Vanessa Vieira, Nina Meurs, Marcel S. Woo, Christian Lohr, Fabio Morellini, Daniela Hirnet, Manuel A. Friese

**Affiliations:** 1 Institute of Neuroimmunology and Multiple Sclerosis (INIMS), University Medical Center Hamburg-Eppendorf, Hamburg, Germany; 2 Institute of Cell and Systems Biology of Animals, University of Hamburg, Hamburg, Germany; 3 Protozoa Immunology, Bernhard Nocht Institute for Tropical Medicine, Hamburg, Germany; 4 Research Group Behavioral Biology, Center for Molecular Neurobiology (ZMNH), University Medical Center Hamburg-Eppendorf, Hamburg, Germany

**Keywords:** Multiple sclerosis, Experimental autoimmune encephalomyelitis, Olfactory bulb, Neuroinflammation, Mitral cells, Single-nucleus RNA sequencing, Monoatomic ion channel activity, Potassium channels, TASK-2

## Abstract

**Background:**

Olfactory dysfunction is an underestimated symptom in multiple sclerosis (MS). Here, we examined the pathogenic mechanisms underlying inflammation-induced dysfunction of the olfactory bulb using the animal model of MS, experimental autoimmune encephalomyelitis (EAE).

**Results:**

Reduced olfactory function in EAE was associated with the degeneration of short-axon neurons, immature neurons, and both mitral and tufted cells, along with their synaptic interactions and axonal repertoire. To dissect the mechanisms underlying the susceptibility of mitral cells, the main projection neurons of the olfactory bulb, we profiled their responses to neuroinflammation by single-nucleus RNA sequencing followed by functional validation. Neuroinflammation resulted in the induction of potassium channel transcripts in mitral cells, which was reflected in increased halothane-induced outward currents of these cells, likely contributing to the impaired olfaction in EAE animals.

**Conclusion:**

This study reveals the crucial role of mitral cells and their potassium channel activity in the olfactory bulb during EAE, thereby enhancing our understanding of neuroinflammation-induced neurodegeneration in MS.

**Supplementary Information:**

The online version contains supplementary material available at 10.1186/s12974-025-03388-5.

## Introduction

Multiple sclerosis (MS) is the most frequent inflammatory disease of the central nervous system (CNS), affecting more than 2.5 million people worldwide [1]. People with MS suffer from a wide range of neurological symptoms, including motor and sensory deficits, such as loss of sensation or vision [2]. Recent evidence suggests that olfactory dysfunction, an often overlooked aspect of the disease, can also significantly affect the quality of life of people with MS [3, 4]. Data suggest a close association between olfactory dysfunction and disease duration and severity [5–7]. It is noteworthy that an elevated olfactory threshold is already detectable in people with clinically isolated syndrome (CIS) [8]. In MS olfactory loss is a commonly seen phenomenon with an olfactory dysfunction up to 84% of patients with primary-progressive MS (PPMS), which affects the olfactory threshold, odor identification and discrimination [9].

In MS, neuronal dysfunction and synaptic and neuroaxonal injury in diverse areas of the CNS are thought to be initiated by aberrantly activated T cells that attack self-antigens of the CNS and cause chronic neuroinflammation [10, 11]. Currently available drugs are only partially effective in slowing the progression of neurodegeneration in MS. Therefore, to halt the progression of neurological disability, it is critical to uncover distinct neuron-intrinsic mechanisms that determine inflammation-induced neurodegeneration in the different CNS regions affected in MS.

Recently, transcriptional profiling by single-cell RNA sequencing or neuron-specific bulk sequencing of MS brains and its model, experimental autoimmune encephalomyelitis (EAE), has revealed pathomechanistically important pathways that are dysregulated at the transcriptional level in neurons [12, 13]. However, the effect of neuroinflammation on the olfactory system at the structural, transcription and functional levels remains unclear, but single-cell transcriptomic profiling gave first insights into the astonishing diversity of neuronal cell types in the olfactory bulb at steady state that could be potentially affected by neuroinflammation [14, 15].

Evolutionary, the olfactory system is one of the oldest sensory systems [16]. Sensory information is processed and integrated in the main olfactory bulb after odors bind to olfactory sensory neurons in the nasal cavity [17, 18]. On histological level, the olfactory bulb can be divided into several layers [19, 20]. Different staining approaches have been used to visualize the morphology of neurons in the different layers more precisely showing morphologically distinct cells in each layer. However, neurons in the olfactory bulb are still categorized based on the layers where their cell bodies reside. This categorization originally defined juxtaglomerular (JG) cells, tufted cells, mitral cells and granule cells. However, it is appreciated that JG cells encompass three morphologically distinct types: periglomerular (PG) cells, external tufted (ET) cells, and superficial short-axon (sSA) cells [21, 22]. More recently, data suggest that each cell population is heterogeneous, each comprising a diverse group of different subpopulations [23]. The projection neurons are composed of mitral and tufted cells, with the latter being defined as external, middle or internal tufted cells, projecting mainly to anterior olfactory regions [24–26]. Mitral cells are of particular interest as they are the predominant output neurons that mainly signal to higher cortical areas, such as the piriform cortex via the lateral olfactory tract [18, 27, 28]. By single-cell RNA sequencing three different types of mitral and tufted cells can be distinguished, but further subdivision was not possible, as they share similar transcriptional profiles and morphological and biophysical properties [14].

Here, we set out to study the impact of neuroinflammation during EAE on the olfactory system at the functional, cellular and molecular level. We detected an impaired odor discrimination in EAE mice, paralleled by immune cell infiltration and neurodegeneration in the olfactory bulb, particularly affecting the tufted and mitral cells. By single-nucleus RNA sequencing (snRNA-seq) we detected changes in mitral cell-specific ion channel expression which coincided with alterations in the outward currents of mitral cells during EAE, explaining impaired olfaction during neuroinflammation.

## Results

### Olfactory dysfunction during EAE

**Fig. 1:**
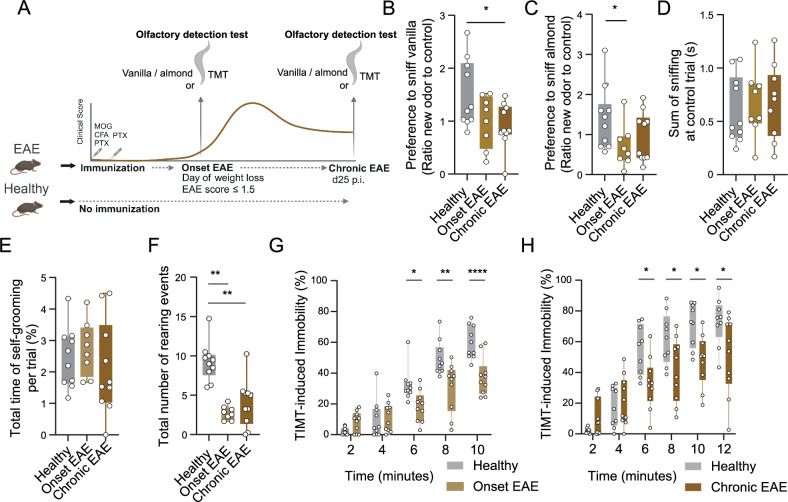
EAE impairs olfaction during the early and chronic phases of the disease. **A** Study design of two different behavioral olfactory tests. Examinations were done at onset of EAE (*n* = 8) and during chronic EAE *(n* = 10) compared to the healthy group (*n* = 10). Visualization done with *Biorender*. **B** Preference to sniff the new odor vanilla during trial 2 of the odor discrimination test in healthy mice (*n* = 10), during onset of EAE (d9 p.i., *n* = 8) and chronic EAE (d25 p.i., *n* = 10). Unpaired, two-tailed Student's t-test was performed for statistical analysis. **C** Preference to sniff the new odor almond during trial 3 in percentage in comparison to a control odor exposed to healthy mice (*n* = 10) as well as at the onset of EAE (d9 p.i., *n* = 8) and during chronic EAE (d25 p.i., *n* = 10). Unpaired, two-tailed Student's t-test was performed for statistical analysis. **D–F** Total time sniffing **D** and self-grooming **E** and number of rearing events **F** in the first trial of the odor discrimination test in healthy mice (*n* = 10), EAE mice at disease onset (d9 p.i., *n* = 8) and during the chronic EAE phase (d25 p.i., *n* = 10). Unpaired, two-tailed Student's t-test was performed for statistical analysis. **G**, **H** Percentage of time the mice were immobile after exposure to 2,5-dihydro-2,4,5-trimethylthiazoline (TMT), measured in 2 min bins, in healthy mice (*n* = 10) and at the onset of EAE (*n* = 10) **G**, as well as in healthy mice (*n* = 9) and in mice during the chronic phase of EAE (*n* = 10) **H**. Graphs show the posthoc test after performance of REML mixed-effects model with Šídák multiple comparison. *P < 0.05, **P < 0.01, ***P < 0.001

To investigate whether the olfactory bulb is functionally affected by neuroinflammation during EAE, we first conducted behavioral tests of olfactory detection using the odor discrimination test with the odors vanilla and almond, as well as a paradigm based on the innate fear response to the odor 2,5-dihydro-2,4,5-trimethylthiazoline (TMT) (Fig. 1A). To control for potential impacts of sickness behavior or hindlimb paralysis in EAE mice on the odor discrimination test, we performed three trials in a cage with two holes at two opposite walls where an odor could be presented. No odor (control condition) was present at both holes during trial 1. In trial 2, we applied vanilla to hole 1 and no odor (control condition) to hole 2. In trial 3, almond was applied to hole 2, with vanilla reapplied to hole 1. To further minimize the impact of major motor deficits on the results, we tested both the onset (day 9 post immunization; d9 p.i.) and chronic stages of the disease (d25 p.i.; the respective clinical scores and weight loss at onset are shown in Fig. S1A, B). EAE mice revealed a significantly reduced preference for the unfamiliar odor vanilla (Fig. 1B) and almond (Fig. 1C) compared to healthy mice. In all groups, the sum of the time sniffing at the holes during trial 1 was comparable (Fig. 1D), indicating that EAE did not generally affect sniffing behavior. Self-grooming, which typically increases during conflictual and stressful conditions, was similar between the groups (Fig. 1E). However, rearing, a typical novelty-induced exploratory behavior, was reduced in diseased mice at EAE onset and in the chronic phase (Fig. 1F). As rearing requires that mice stand on their hindlimb, motor deficits could have impacted this behavior.

To confirm the impaired olfactory novelty detection in EAE mice, we performed a second olfactory test using the aversive odor TMT that induces immobility in mice.[29]. After exposure to the TMT odor, mice suffering from EAE exhibited significantly reduced immobility response to TMT than healthy control mice in both the early stage (d10 p.i.) and late stage (d25 p.i.) of the disease (Fig. 1G, H). Due to possible influences of the animals' motor deficits, we deliberately analyzed these mice not at the peak of the disease, but at the onset and during the chronic phase of EAE (see also Fig. 1A). The effects on rearing in these tests were similar to those observed in the previous test (Fig. S1C, D). Our data suggest that a profound impairment in odor detection is already present at the early EAE disease stage and remains impaired during chronic stages.

### Immune cell infiltration in the olfactory bulb during EAE

**Fig. 2:**
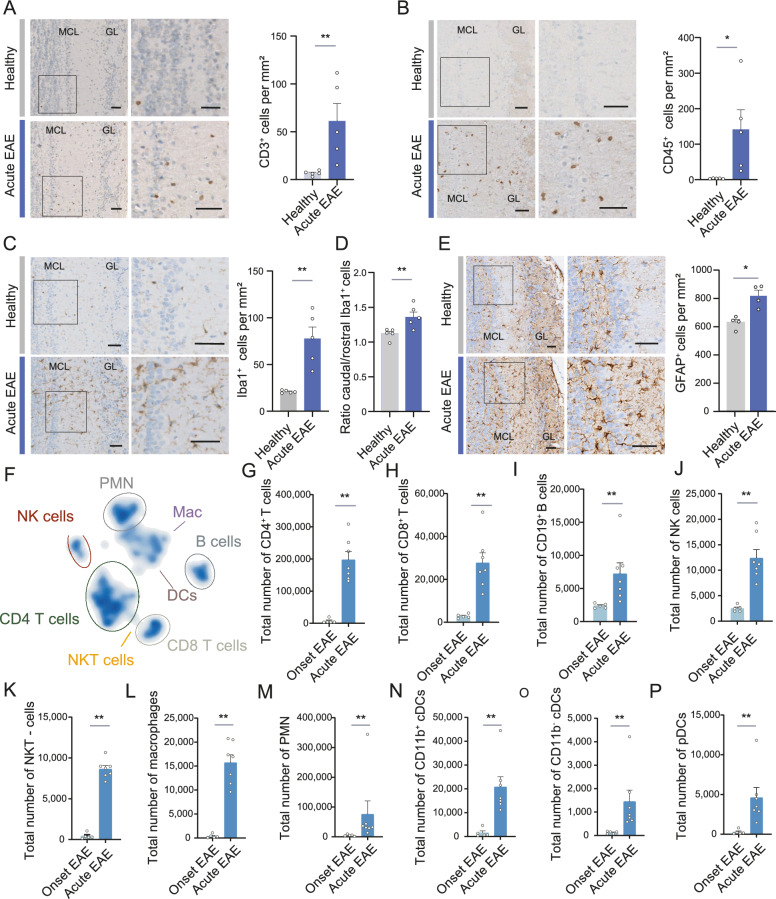
Immune cell infiltration and glial cell activation in the olfactory bulb during EAE. **A–C** Representative image with zoom-in (enlarged section displayed by frame in overview image) and quantification of **A** CD3^+^ cells infiltrating, (B) CD45^+^ macrophages and (C) Iba1^+^ microglia in the main olfactory bulb (coronar orientation) in healthy mice and at the acute phase of EAE (d15 p.i.) (*n* = 5 per group). Scale bar = 50 µm. **D** Localization of microglial activation measured by the ratio of microglial cell number per mm^2^ in the lower caudal part of the olfactory bulb compared to the upper cranial part of the olfactory bulb in healthy mice and at the acute phase of EAE (*n* = 5 per group). **E** Representative images with zoom-in (enlarged section displayed by frame in overview image) and quantification per mm^2^ of GFAP^+^ astrocytes in the olfactory bulb of healthy mice and at the acute phase of EAE (*n* = 5 per group). Scale bar = 50 µm. **F** UMAP plot showing the distribution of different immune cells infiltrating the olfactory bulb during the acute phase of EAE measured by flow cytometry (*n* = 12). **G–P** Quantification of immune cells isolated from olfactory bulbs at the onset of EAE (d9 p.i., *n* = 5) and at the acute phase of EAE (d15 p.i., *n* = 7) measured by flow cytometry. Shown are CD4^+^ T cells (**G**) and CD8^+^ T cells (**H**), CD19^+^ B cells (**I**), NK cells (**J**), NK-T cells (**K**), macrophages (**L**), polymorphonuclear neutrophiles = PMN (**M**), CD11b^+^ (**N**) and CD11b^–^ (**O**) conventional dendritic cells = cDC, plasmacytoid dendritic cells = pDC (**P**). Bars show mean values ± s.e.m. Statistical analysis was performed by Mann–Whitney U-Test; *P < 0.05, **P < 0.01

To investigate the mechanisms underlying the olfactory dysfunction, we characterized the cell type composition in the olfactory bulb during neuroinflammation. We started by recording the immune cell infiltration in the olfactory bulb by using immunohistochemistry and flow cytometry. A significant increase in both CD3^+^ T cells (Fig. 2A) and CD45^+^ leukocytes (Fig. 2B) was detected in the main olfactory bulb during the acute phase of EAE (d15 p.i.). Additionally, microglia activation was assessed by measuring the number of ionized calcium-binding adapter molecule 1 protein IBA1^+^ cells per area during the acute phase (Fig. 2C). This was further confirmed by staining of two other microglial markers: P2Y12, which indicates a quiescent microglial state, showed decreased expression during inflammation (Fig. S2A), while the transmembrane protein 119 (TMEM119), a predominant marker of reactive microglia, was upregulated during acute EAE (Fig. S2B). Microglial activation was more pronounced in the caudal than in the rostral part of the olfactory bulb (Fig. 2D). Microglial activation as well as this distribution pattern persisted in the chronic phase of the disease at d30 p.i. (Fig. S2C–D). In parallel with activation of microglia and immune cell infiltration we detected an increased number of GFAP^+^ cells. (Fig. 2E). Additional flow cytometry-based evaluation of the immune cell subtypes infiltrating the olfactory bulb in the early and acute phase revealed a broad immune cell composition which is visualized by a UMAP plot (Fig. 2F). In addition to T cells and B cells, natural killer (NK) cells were already detectable at the onset of EAE (d9 p.i.) (Fig. 2G–P). At the peak of disease (acute EAE; d14 p.i.), next to CD4^+^ and CD8^+^ T cells, B cells and NK cells, a significant increase in all measured innate immune cells (including polymorphonuclear neutrophils (PMN), macrophages, conventional dendritic cells (cDC), plasmacytoid dendritic cells (pDC), and NKT cells) was detectable. However, this was dominated by CD4^+^ T cells (Fig. 2G–P and Fig. S2E). The immune cell composition in the olfactory bulb did not fundamentally differ from that in the spinal cord at the same time points, as visualized by a UMAP plot (Fig. S2F). However, when comparing the frequencies of immune cells between these tissues, macrophages and NK cells as well as NKT cells were significantly more frequent in the population of CD45hi-expressing cells in the olfactory bulb compared to the spinal cord at the peak of the disease. In contrast, neutrophiles and CD11b-negative cDCs were more prominent in the spinal cord (Fig. S2G–P).

Overall, our data show that, beyond the spinal cord, a broad immune cell infiltration can also be detected in the main olfactory bulb during the early and acute phases of EAE.

### Neuronal characterization of inflammation-induced neurodegeneration

**Fig. 3:**
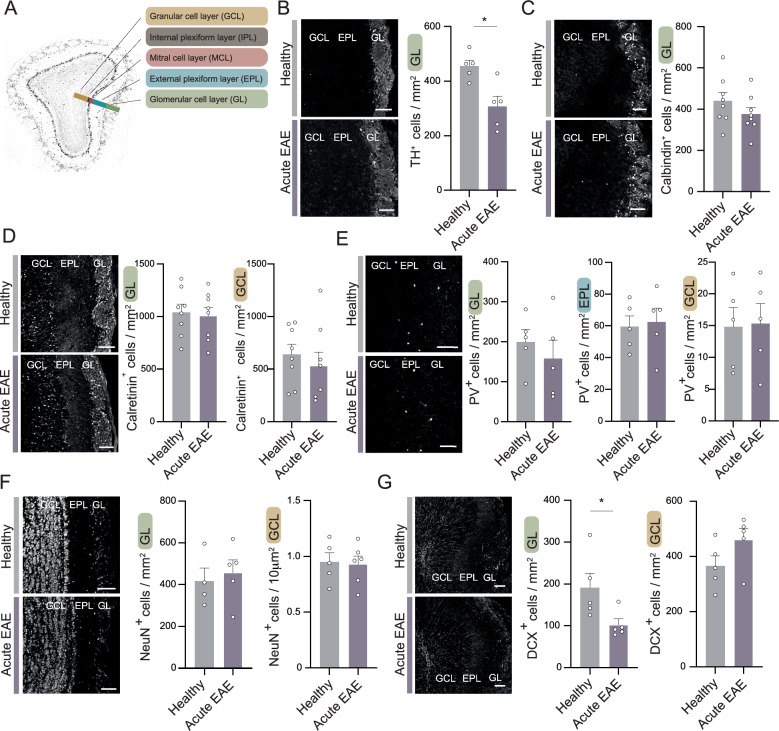
Structural analyses show cell-type specific neuronal vulnerability during EAE. **A** Representation of the most prominent layers in the main olfactory bulb. Glomerular layer = GL, external plexiform layer = EPL, mitral cell layer = MCL, internal plexiform layer = IPL, granular cell layer = GCL. **B–G** Representative immunofluorescence images and quantitative measurement of different cell populations according to their main cell markers measured in the olfactory bulb per mm^2^ in healthy and EAE mice (*n* = 5–8 per group): **B** Tyrosine hydroxylase (TH), **C** Calbindin, **D** Calretinin, **E** Parvalbumin (PV), **F** NeuN and **G** Doublecortin (DCX). The analyzed layers are described and colored according to the representative image in **A**. Scale bars = 100 µm. Bars show mean values ± s.e.m. Statistical analysis was performed by unpaired, two-tailed Student's t-test; *P < 0.05

Next, we characterized the impact of the immune cell infiltration and activation on neurodegeneration in the olfactory bulb during EAE. We analyzed key neuronal cell populations by immunohistochemistry during healthy and EAE conditions in the glomerular layer (GL), external plexiform layer (EPL), mitral cell layer (MCL) and granular cell layer (GCL). An overview of the main layers is shown in Fig. 3A. In acute EAE (d15 p.i.) three neuronal cell subtypes were significantly reduced in the main olfactory bulb. Among the broad subset of mainly inhibitory neurons, the tyrosine hydroxylase (TH)-positive cells in the GL were significantly reduced under EAE conditions Fig. 3B). However, other inhibitory populations expressing calbindin (Fig. 3C), calretinin (Fig. 3D) or parvalbumin (Fig. 3E) were not affected in the GL, EPL or GCL. No significant inflammation-induced loss was visible in NeuN-positive neurons, which are mainly defined as mature neurons, across the different layers of the olfactory bulb (Fig. 3F). Cells expressing the marker doublecortin (DCX), indicating immature neurons, were also significantly reduced in the GL, but not in the GCL at the peak of EAE disease (Fig. 3G). DCX-positive cells represent immature interneurons, which are part of the pool of neurons migrating from the subventricular zone via the rostral migratory stream to the olfactory bulb. This dynamic modulation suggests a complex response to the neuroinflammatory condition. Therefore, we further analyzed the impact of prolonged neuroinflammatory exposure at the chronic stage of EAE (d30 p.i.). Here, DCX-positive cells were significantly reduced in both analyzed compartments, the granule cell layer and the glomerular cell layer (Fig. S3A).

**Fig. 4:**
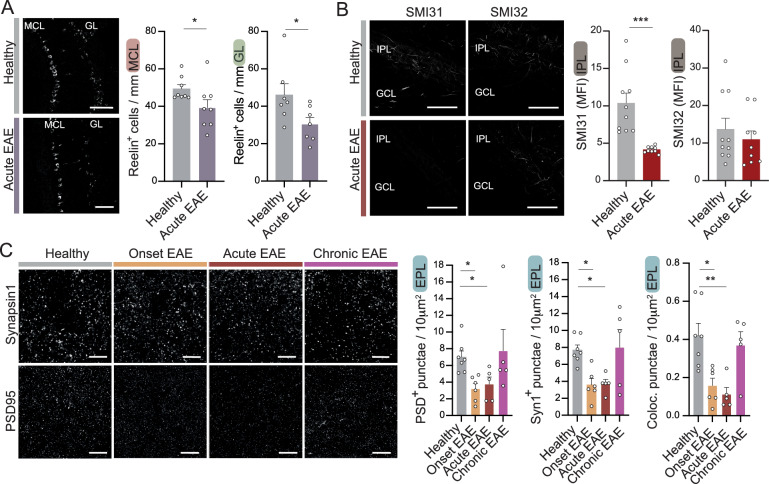
Mitral cells are susceptible to neuroinflammation in EAE. **A** Representative immunofluorescence image and quantification of reelin-positive mitral cells per mm mitral cell layer (MCL) and glomerular layer (GL) in healthy mice and mice at acute EAE (d15 p.i.) (MC: *n* = 8 per group; GL: *n* = 7 per group). Scale bars = 100 µm. **B** Representative images and quantification of phosphorylated (SMI31) and non- phosphorylated (SMI32) neurofilaments measured by mean fluorescence intensity (MFI) in the internal plexiform layer (IPL) of healthy (*n* = 10) and EAE animals (*n* = 9 per group). Scale bars = 100 µm. **C** Visualization and quantification of the synaptic density in the external plexiform layer measured by positive particles per area in mm^2^ of postsynaptic density protein PSD95 and presynaptic protein synapsin 1 (Syn1), as well as the colocalization of both, with latter corresponding to synapses between mitral cells and granular cells, in healthy animals, in onset EAE (d10 p.i.), acute EAE (d15 p.i.) and chronic EAE (d30 p.i.) (*n* = 5–7 per group). Scale bars = 20 µm. Bars show mean values ± s.e.m. Statistical analysis was performed one-way ANOVA followed by Tukey’s post hoc test; *P < 0.05, **P < 0.01, ***P < 0.001

Within the excitatory neuronal populations, the most prominent are the mitral cells which are the main projection neurons of the olfactory bulb. These cells also proved susceptible to the neuroinflammatory exposure. Reelin-positive cells, which mark mitral cells in the mitral cell layer and tufted cells in and adjacent to the glomerular cell layer, were significantly reduced in their respective cell layers during the acute stage (Fig. 4A) and in the mitral cell layer during the chronic stage of EAE (Fig. S4A). We confirmed the significant reduction of reelin-positive mitral cells during EAE in a second cohort of animals by quantifying highly HuC/D-positive cells in the mitral cell layer during the time course of EAE (EAE d10 p.i., EAE d15 p.i., EAE d30 p.i.) and in healthy mice (Fig. S4B). HuC and HuD are members of the Elav family of RNA-binding proteins, specifically expressed in neuronal cells, making them useful markers for identifying neuronal cells [30].

To gain further insights into the functional disturbance of mitral cells, we analyzed axonal pathology by immunohistochemical staining of healthy phosphorylated (SMI31) and injured non-phosphorylated neurofilament H (SMI32) expressing axons in the inner plexiform layer, where axons of mitral cells are localized in their projection to higher cortical areas. Significant reduction was seen in SMI31 but not in SMI32 (Fig. 4B). Axonal pathology was accompanied by a significant reduction in myelin basic protein (MBP) in the inner plexiform layer at the acute stage of EAE (Fig. S4C). Integration of olfactory information, among other processes, occurs in reciprocal synaptic connections between mitral cells and granule cells. During acute EAE, synaptic density is affected in the external plexiform layer in the olfactory bulb, as measured by the postsynaptic protein PSD95 and the presynaptic protein synapsin1 and their colocalization (Fig. 4C). However, in the chronic phase of EAE, synaptic count appears to recover (Fig. 4C), despite the loss of a portion of the mitral cells described above (Fig. S4A, S4B).

These results confirm the impact on several neuronal cell types and their projections in the olfactory bulb during EAE.

### Single-nucleus RNA sequencing of HuC/D^+^ olfactory bulb cells

**Fig. 5:**
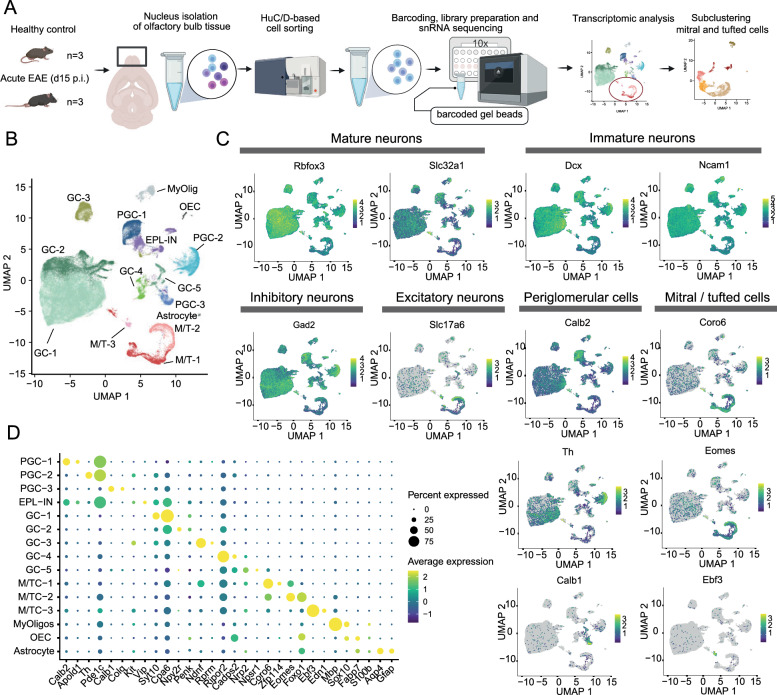
SnRNA-Seq of the olfactory bulb reveals a heterogeneity of neuronal cell types. **A** Study design of single-nucleus RNA sequencing approach of neuronal nuclei in the olfactory bulb of healthy and acute EAE mice. Visualization done with *Biorender*. **B** UMAP plot with a total of 73,835 cells from six mice revealing 15 different clusters of mostly neuronal cells in the olfactory bulb. PGC = periglomerular cells, M/T = mitral/tufted cells, GC = granular cells, EPL-IN = interneurons of the external plexiform layer, MyOligos = myelinating oligodendrocytes, OEC = olfactory ensheathing cells. **C** Feature plots of prominent cell types including mature neurons, immature neurons, inhibitory neurons, excitatory neurons and two groups of prominent cell populations as defined by the depicted marker gene: Periglomerular cells with calretinin(Calb2)-positive, TH-positive and calbindin(Calb1)-positive cells as well as mitral/tufted cell clusters, which are further analyzed in Fig. 6. **D** Dot plot of the marker genes of the 15 distinct clusters

To further understand the underlying pathomechanisms of inflammation-induced neurodegeneration in various neuronal cell types, we performed single-nucleus RNA sequencing (snRNA-seq) of the olfactory bulb under healthy and inflamed conditions. Our focus was primarily on mitral cells, which were significantly affected in our histological analyses. Instead of using the established neuronal marker NeuN for neuronal nuclei – since NeuN is barely expressed in certain neuronal cell types including mitral cells and Purkinje cells [31] – we employed HuC/D staining. This allowed us to isolate all relevant neuronal nuclei of the olfactory bulb, including the mitral cell compartment, via flow cytometry sorting (schematic workflow shown in Fig. 5A; flow panel in Fig. S5A). Validation experiments confirmed neuronal enrichment (as indicated by the expression of *Cdhr1, Rbfox3, Grin1, Dcx*) with a significant enrichment of the mitral cell-specific gene *Cdhr1* and a strong de-enrichment of non-neuronal cell populations in the sorted samples (measured by the expression of* Gfap, Mbp* and* Cxcr1*, see Fig. S5B).

Isolated nuclei of three healthy and three EAE mice at the acute stage (d15 p.i.) were FACS-sorted using the HuC/D marker and profiled by snRNA-seq. Seurat-based clustering of the nuclei revealed 15 different clusters (Fig. 5B). Cell count and quality parameters are presented in Table S2. The differentiation of clusters is shown as heatmap of marker genes in Fig. S5C. Given the sparsity of single-cell and single-nuclei datasets for olfactory bulb neurons, clusters were manually assigned to the cell populations. We identified 15 different clusters: three M/T clusters, three clusters of periglomerular cells (PGCs), of which one most likely consists of short-axon cells due to their TH positivity; one cluster of interneurons belonging to the EPL (EPL-IN); six clusters of granule cells (GC); one cluster consisting of cells of the oligodendrocyte lineage (MyOlig); one small cluster which can be assigned to the olfactory ensheathing cells (OEC) and one astrocytic cluster (Astro). Overall, a heterogeneity of neuronal cells was detected. The feature plots in Fig. 5C visualize the distribution of predominantly mature neurons (*Rbfox3* and *Slc32a1*), and more immature neurons (represented by *Dcx* and *Ncam1*), overall inhibitory neurons (by *Gad2*) and excitatory neurons (visualized by *Slc17a6*). The PGC populations included three clusters, with PGC-1 showing high expression of *Apold1* and *Calb2*. However, *Calb2* was not exclusive to this cluster but included some granule cells as observed in the immunohistochemical stainings. High TH positivity and *Pde1c* expression were detected in the PGC-2 cluster, and the PGC-3 was characterized by *Calb1* expression. A cluster of EPL interneurons presented a unique subset of marker genes, with expression of *Vip, Sst, Pvalb,* and *Kit.* The granule cells exhibited fewer unique markers as seen in Fig. 5D, where selected genes classified three mitral/tufted cell clusters (M/TC-1 with the highest expression of *Coro6* and *Zfp114*; M/TC-2 with the highest expression of the genes *Eomes* and *Foxo1;* and the small cluster M/TC-3 which highly expressed the marker *Ebf3* and *Edn1*).

The granule cells exhibited fewer unique marker genes, as seen in Fig. 5D, where selected genes for discrimination of all cell clusters are presented. Three non-neuronal cells were identified: a cluster (labeled MyOligo) expressing *Mbp* and *Mog*, indicating an oligodendrocytic origin with rather differentiated cell characteristic; another cluster, which comprised a more ambiguous cell cluster with expression of *Sox10* and *S100b,* possibly indicating olfactory ensheathing cells, and a final cluster expressing the classic astrocytic marker *Aqp4*. Additional marker genes of interest are visualized in Fig. S5D. Overall, snRNA-seq enabled us to characterize the transcriptomic landscape of the diverse neuronal cell populations in the olfactory bulb.

### Ion channel expression changes in mitral cells during EAE

**Fig. 6:**
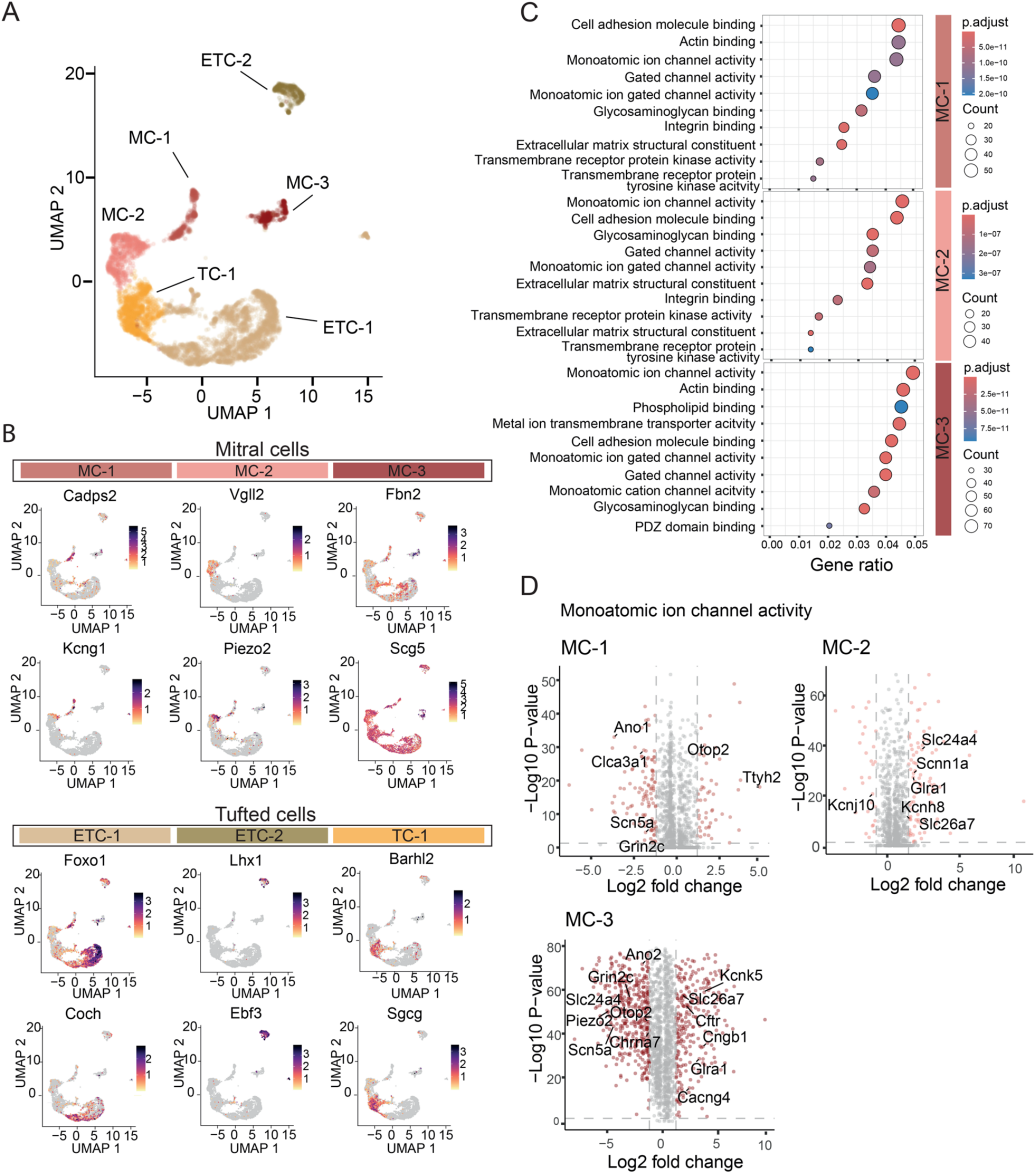
SnRNA-Seq analysis reveals expression changes related to monoatomic ion channel activity in inflamed mitral cells **A** UMAP plot of the three mitral/tufted cell clusters filtered from the main clusters as depicted in Fig. 5. A total of 7,252 cells grouped in six distinct clusters with three mitral cell clusters (MC-1, MC-2, MC-3), two external tufted cell clusters (ETC-1, ETC-2) and one middle tufted cell cluster (TC-1). **B** Feature plots of selected marker genes of mitral and tufted cells based on published data [15]. **C** Dot plot of the gene ontology enrichment analysis (GO term analysis) of the three mitral cell clusters. **D** Volcano plots of the differentially expressed genes of mitral cell clusters MC-1, MC-2 and MC-3 of healthy mice compared to acute EAE. Significantly regulated genes of the GO term “monoatomic ion channel activity” are labeled

To take a closer look at the mitral cells, we further analyzed the three M/T clusters. By further zooming into possible subclusters of the 7,252 M/T nuclei (healthy *n* = 3,773, acute EAE *n* = 3,479), we identified three mitral cell clusters (MC-1, MC-2, MC-3) and three tufted cell clusters, with two of them corresponding to external tufted cells (ETC-1, ETC-2) and one to a population of middle tufted cells (TC-1) (Fig. 6A, Fig. S6A). The assignment of the mitral and tufted cells was done on the basis of a previous dataset [15]. The important marker genes to distinguish the six clusters are presented by UMAP plots in Fig. 6B. Selected markers for mitral cells included *Cadps2, Kcng1, Vgll2, Piezo2, Fbn2* and *Scg5*. MC-1 and MC-3 showed partially overlapping marker gene expression. Tufted cell marker genes selected according to a previously published dataset [15] were *Foxo1, Coch, Lhx1, Ebf3, Barhl2,* and *Sgcg*. The marker *Ebf3*, highly expressed in ETC-2, was already selective for cluster 12 in the main dataset. Other markers, such as the gene *Olfr111,* were present in more than one cluster (see Fig. S6B).

Since neuronal cell populations were not significantly reduced during neuroinflammation compared to healthy mice, in this dataset of three versus three mice (Fig. S6C), we evaluated more subtle changes in the inflamed olfactory bulb. The clusters MC-2 and MC-3 showed a reduction in absolute cell numbers in acute EAE compared to healthy mice. This was not seen in the tufted cell clusters and supported our further emphasis on the analysis of the mitral cell clusters. By analyzing differential gene expression of mitral cells, which is presented in Fig. S6D, we observed among others, an induction of genes associated with interferon responses (*Gbp6*) and related down-stream signaling molecules (*Lef-1*). Additionally, several ion channels were upregulated in EAE mice, including *Ttyh1* and *Kcnk5*. To further evaluate the relevance of these observations and to assess similarities between mitral cell populations, we conducted gene ontology (GO) term enrichment analyses of the mitral cell clusters. The GO term ‘monoatomic ion channel activity’ was highly associated with any mitral cell cluster (Fig. 6C). Additionally, the related GO terms ‘monoatomic ion gated channel activity’, ‘gated channel activity’, and ‘monoatomic cation channel activity’ were common among the clusters. Further, we analyzed the genes included in these GO terms for differential gene expression (Fig. 6D). In inflamed mitral cells of cluster MC-1, the genes of the chloride channel *Ttyh2* and the proton channel *Otop2* were significantly induced; in cluster MC-2, the genes included sodium/potassium/calcium exchanger 4 (*Slc24a4*), sodium channel (*Scnn1a*), glycine receptor 1 (*Gira*), and anion exchange transporter (*Slc26a7*) were upregulated; and in cluster MC-3, the genes of the potassium channels K_2P_5.1, encoded by *Kcnk5*, anion exchange transporter (*Slc26a7*), chloride channel (*Cftr*), cGMP-gated cation channel (*Cngb1*), and voltage-dependent calcium channel gamma-4 (*Cacng4*) were noted to be upregulated. We validated gene expression changes of selected genes by qPCR in a separate cohort of healthy and acute EAE samples, focusing on HuC/D^high^ nuclei enriched for mitral cells (Fig. S6E–G).

Together, the transcriptomic analysis of the mitral cell clusters revealed ion channel perturbations during the acute stage of EAE.

### Functional perturbation of mitral cells during neuroinflammation

**Fig. 7:**
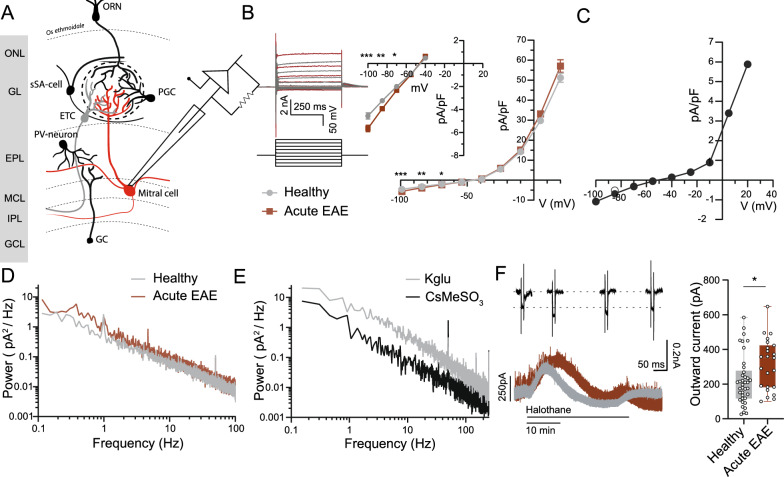
Electrophysiological properties of mitral cells are perturbed during EAE. **A** Schematic presentation of whole-clamp in mitral cells of the main olfactory bulb. Olfactory receptor neuron = ORN; olfactory nerve layer = ONL; glomerular layer = GL; external plexiform layer = EPL; mitral cell layer = MCL; internal plexiform layer = IPL; granule cell layer = GCL; superficial short axon cell = sSA; periglomerular cell = PGC; external tufted cell = ETC; parvalbumin = PV-positive neuron; granule cell = GC. **B** Whole-cell currents recorded upon voltage-steps from –100 mV to 20 mV (step size 15 mV), were normalized by cell capacitance to compare current density of mitral cells in healthy (*n* = 39 cells, n = 25 animals) and acute EAE (d13–16 p.i., *n* = 43 cells, *n* = 20 animals). Inset shows increased inward current at negative potentials. Statistics were done by Mann–Whitney U-test; *P < 0.05, **P < 0.01, ***P < 0.001. **C** Difference in pA/pF throughout the I/V curve between healthy and inflamed mitral cells of the same cohort. **D** Power density spectrum (in pA^2^/Hz) in mitral cells of healthy (*n* = 38 cells, *n* = 30 animals) vs. acute EAE (d13–16 p.i., *n* = 23 cells, *n* = 12 animals). Statistical analysis was performed with two-sampled Kolmogorov–Smirnov test. **E** Power spectral density in pA^2^/Hz in healthy mitral cells with potassium-based intracellular solution (potassium glutamate = Kglu; *n* = 10 cells, *n* = 7 animals) or cesium-based intracellular solution (CsMeSO_3_; *n* = 10 cells, *n* = 2 animals). Statistical analysis was performed with two-sampled Kolmogorov–Smirnov test. **F** Representative traces and quantification of halothane-induced current (pA) in mitral cells of healthy (*n* = 38 cells, *n* = 30 animals) and EAE (*n* = 23 cells, *n* = 12 animals). Statistical analysis was performed by unpaired, Mann–Whitney U-test**;** *P < 0.05

Given the expression changes of several ion channels in mitral cells during neuroinflammation, we decided to explore their integrated functional impact in whole-cell patch-clamp recordings. We investigated mitral cells via patch-clamp electrophysiology in brain slices from healthy and EAE animals to probe physiological alterations during neuroinflammation (Fig. 7A). After patching the mitral cells, a voltage step protocol was applied, and the resulting whole-cell currents were recorded to examine the current–voltage (I/V) relationship. The cell capacitance of both groups was similar in healthy (71.1 ± 2.8 pF; *n* = 39) and in EAE animals (70.4 ± 10.7 pF; *n* = 43; *P* = 0.782; Fig. S7A) as was the resting membrane potential (−45.6 ± 0.7 mV; *n* = 39, versus −46.2 ± 0.8 mV; *n* = 43; *P* = 0.638, measured in DOWN state immediately after establishing of whole-cell configuration; Fig. S7B). However, the I/V relationship of the whole-cell currents in inflamed olfactory bulbs showed a significantly increased inward current at negative potentials compared to healthy olfactory bulbs (healthy *n* = 39 cells, n = 25 animals and EAE *n* = 43 cells, *n* = 20 animals; −100 mV, *P* = 0.0003; −85 mV, *P* = 0.0012; −70 mV, *P* = 0.024; Fig. 7B). To reveal the current that changes under inflammatory conditions, we subtracted the I/V plots of both conditions. The I/V plot of the estimated EAE-induced current showed the typical shape of potassium conductance with a negative reversal potential and outward rectifying behavior (Fig. 7C).

The increase in conductance at negative potentials may be due to elevated I_h_ currents in mitral cells under inflammatory conditions. Mitral cells express several HCN channel subtypes, which regulate biphasic membrane potential and burst firing [32, 33] We recorded whole-cell currents from mitral cells using a voltage step protocol, revealing the slow activation at negative potentials typical of I_h_ (Fig. S7C). However, we found no significant difference in I_h_ current between mitral cells from healthy and EAE animals, and single-nucleus sequencing data showed no differential HCN expression under inflammatory conditions.

To further investigate the changes under inflammatory conditions, we analyzed the fluctuations of holding current in voltage clamp as a surrogate of subthreshold voltage fluctuations in the cell [34]. We analyzed the power spectral density (PSD) of the current traces of mitral cells (Fig. 7D), focusing on signal frequencies in the range of 0.1 to 100 Hz, which covers the main part of signals of biological origin. Mitral cells showed the typical shape of single-cell PSD as demonstrated previously [35], with the highest power at low frequencies and a negative linear slope in the log–log domain. Comparing the PSD from healthy and EAE animals, we observed an upward shift of the PSD curve in EAE mice, which was pronounced in the lower frequency range. A similar difference in the power of the PSD was found in healthy animals between recordings with low and high K^+^ conductance, suggesting that the increase in the PSD in EAE animals might be caused by an increase in K^+^ conductance (Fig. 7E). In these recordings, synaptic inputs in mitral cells were blocked to isolate the effect of the intrinsic K^+^ conductance on the PSD and minimize the impact of the neuronal network. To quantify the difference in mitral cell membrane current oscillations between healthy and inflamed conditions, we calculated the average power as the integral of the corresponding linear spectrograms (Fig. S7D). This analysis revealed an overall increase in oscillation power in diseased mitral cells (5.9 pA^2^, *n* = 23) compared to healthy cells (3.6 pA^2^, *n* = 38, *P* = 0,025) with a significant increase in the delta frequency band (3.0 vs. 1.5 pA^2^, *P* = 0.016) (Fig. S7D, corresponding visualization of the delta band in Fig. S7E).

Inferring from the results of the whole-cell current and PSD analysis, and based on the differential expression data obtained by snRNA-seq, potassium channels of the 2-pore domain potassium channel (K2P) subfamily might be primary candidates for the potassium conductance affected by EAE. For recording potassium currents driven by K2P channels, we used the anesthetic halothane, known to activate a subset of K2P channels and inhibit others, and investigated the induced currents in mitral cells from both healthy control animals and EAE animals. Upon application of halothane, the mitral cells showed a biphasic response (Fig. 7E), with an initial outward current concomitant with an increase of conductance, followed in some cells by an inward current of variable amplitude. On average, the amplitude of the halothane-induced outward current of mitral cells of healthy control animals (229 pA ± 23, *n* = 38 cells, *n* = 30 animals) was smaller than in cells of EAE animals (314 ± 30 pA, *n* = 23 cells, *n* = 12 animals, *P* = 0.027), suggesting an involvement of halothane-sensitive K2P channels in the pathological changes of mitral cells under neuroinflammatory challenge.

In summary, perturbation of potassium channel physiology in mitral cells is associated with olfactory dysfunction during neuroinflammation.

## Discussion

Inflammation-induced neurodegeneration is a hallmark of MS, which is also reflected by olfactory dysfunction. We comprehensively investigated the cellular mechanisms underlying dysfunctional olfactory perception in a mouse model of MS.

First, we aimed to evaluate changes in olfaction during EAE, a mouse model of MS. Interestingly, in two olfactory detection tests, we observed that in mice immunized with MOG develop olfactory dysfunction at onset of disease and at chronic stage of disease, similar to what has been described in MS [3, 8, 36–38], but also in our EAE mouse model through two olfactory detection tests. Motor deficits and sickness behavior are critical confounders in motor-based olfactory tests, such as the buried food test [39], which we also observed in our experiments by the decreased rearing events. Furthermore, different subcomponents of olfactory function—identification, discrimination, and threshold—may be affected differently at various stages of the disease. Our two-sided detection test, using vanilla and almond as test odors, primarily addressed threshold and discrimination. The TMT-based detection test is more sensitive for assessing olfactory threshold. In people with MS, disturbed olfactory threshold correlates with relapse activity [7, 36]. Interestingly, in both tests, olfactory function was already disturbed at the onset of EAE, suggesting that subtle inflammation already affects olfactory function.

Next, we wanted to investigate the mechanisms that underly the olfactory dysfunction we observed. To this end, we correlated olfaction to neuroinflammation and neurodegeneration in the olfactory bulb during EAE. The predominant immune cells were T cells, specifically CD4^+^ T cells and neutrophils. To date, extensive evidence supports the critical role of T cells, particularly CD4^+^ T cells, in MS pathophysiology [11]. Additionally, neutrophils have been hypothesized to contribute to the pathogenesis of EAE by producing cytokines and facilitating the disruption of the blood brain barrier [40]. Additionally, neutrophils influence the manifestation of EAE by facilitating antigen-presentation and parenchymal brain inflammation [41, 42]. The region-specific differences between the olfactory bulb and spinal cord, notably the increased frequency of NK cells, NKT cells and macrophages, warrant further investigation by selectively analyzing these cell populations and their tissue-specific cues of recruitment. For example, the role of NK cells may vary based on their CD56 expression level and location, necessitating further characterization [43–46]. However, only limited conclusions can be drawn regarding the immune cell repertoire in the olfactory bulb of people with MS and a comprehensive characterization of the olfactory bulb in MS has not been performed so far. Although only few cells (30,410 ± 8,784 CD45^high^ cells) were detected infiltrating the olfactory bulb at the onset of EAE, we were already able to observe olfactory dysfunction in behavioral tests at this early stage. Neuronal function may be already affected by cytokine release from immune cells [47]. However, further studies are needed to investigate this aspect.

By utilizing immunohistochemistry to analyze the impact of inflammation, we identified the main projection neurons of the main olfactory bulb, the mitral cells, along with TH-positive and DCX-positive neurons, as being particularly susceptible to neuroinflammation. Furthermore, we confirmed axonal pathology in mitral cells via neurofilament staining, and a significant reduction in phosphorylated neurofilament. Phosphorylated neurofilament, detected by SMI31, is typically localized in axons under physiological conditions but can shift to the cell soma during injury [48]. The inner plexiform layer analyzed here primarily contains mitral cell axons with few somata, so the observed reduction of SMI31 aligns with previous studies [49, 50]. SMI31 reduction has been reported not only in inactive but also in active lesions, indicating modulation even in the acute inflammatory state [51]. The early involvement of the mitral cell network, including synaptic density in the external plexiform layer (Fig. 4E), may contribute to the early SMI31 reduction during EAE in the olfactory bulb.

The mitral cell, the projection neuron of the olfactory bulb, integrates sensory inputs and local inputs from interneurons, eventually forming the odor code transmitted to and interpreted by higher-order brain areas such as the piriform and entorhinal cortex [52]. Given the critical dependence of this odor code on the precise local and temporal firing of mitral cell groups, maintaining the functionality of these cells is vital for olfactory perception [53]. Aware of the pivotal role of mitral cells, we assessed them in a more detailed analysis using snRNA-seq. This method enabled us to specifically investigate inflammation-dependent perturbation in the small mitral cell population and the diverse neuronal landscape of the olfactory bulb, in contrast to recent publications that performed bulk analyses of the entire olfactory bulb, including neurons, glial cells, and infiltrating immune cells [54, 55].

The unbiased analysis of the transcriptional signatures of olfactory neuronal cells revealed a heterogeneity of neuronal cells in the olfactory bulb with a high number of interneurons corroborating previous analyses of the main olfactory bulb [14, 56]. Unlike the periglomerular cells, interneurons of the external plexiform layer, and the mitral/tufted cells, the granular cell clusters were less distinctly identifiable by marker genes, consistent with earlier reports of some clusters lacking defined cell type-specific marker genes [14]. The transcriptional uniqueness may stem from the migration of adult-born neurons from the subventricular zone to the main olfactory bulb, continuing their radial migration to the glomerular area [57]. This dynamic cellular state could account for the detected co-expression of markers typical for immature neuron, *Rbfox3*, which encodes for NeuN, and DCX [14, 58]. Using the nuclear marker HuC/D allowed us to focus our analysis on the primary cell type of interest, the mitral and tufted cells. However, we also identified some non-neuronal cells, mainly SOX10-positive nuclei from the oligodendrocyte lineage, as previously reported in human studies [30] (see also Fig. S5).

Focusing on the mitral cell clusters in the single-cell data set of healthy and EAE mice we were able to detect the alteration of monoatomic ion channel activity in the EAE condition. In the three mitral cell clusters several genes related to ion channels were upregulated, mainly chloride (*Ttyh2, Glra1)* and potassium (*Kcnk5, Kcnh8, Slc24a4)* channels or exchangers. After comprehensive functional validation potassium channel activity was perturbed most striking on the functional level in the inflamed mitral cells. Potassium channels regulate every aspect of the neuronal function, from resting membrane potential to excitability and firing pattern, and are therefore key factors for neuronal functionality. There are more than 70 known genes coding for potassium channels in mammals, giving rise to K^+^ channel proteins with different primary structure and subunit compositions [59]. K2P channels are voltage-independent leak channels active at negative membrane potentials, accounting for the resting membrane potential of neurons. They are also involved in regulating excitability and controlling firing [60]. However, changes in pH and the release of pro-inflammatory cytokines such as interleukin-17 or interferon-γ during inflammatory conditions might also alter the activity of K2P channels [61]. Indeed, in neuroinflammation, a change in the expression and function of K2P channels has been reported [62, 63]. In the olfactory bulb, several subtypes of K2P channels are expressed [64–67]. Anesthetics such as halothane are often used to discriminate different K2P subfamilies functionally [68, 69]. In our experiments, halothane induced a biphasic response in mitral cells suggesting the involvement of more than one type of halothane-sensitive channel in mitral cells. Nonetheless, the outward component of the halothane response was increased in EAE animals. By increasing the leak conductance, the mitral cells might respond to depolarizing and thereby excitotoxic conditions such as increased glutamatergic release in the neuronal network, altered glutamatergic currents, and acidification [70–73].

Enhanced potassium efflux may initially prevent overexcitation by inducing hyperpolarization, shunting inhibition, and reducing excitability. However, this is offset by impaired action potential propagation, worsening neurological symptoms [74]. In the long term, high potassium channel expression can be harmful. Extracellular potassium buildup may disrupt electrical homeostasis, while intracellular depletion could trigger apoptotic pathways [75]. Although K2P channel expression seems protective against neuronal degeneration in cerebral ischemia [63], its role in neuroinflammation remains unclear and requires further investigation.

We analyzed noise properties in the electrophysiological recordings, as an additional approach to reveal physiological changes in cell and circuit dynamics. Analyzing noise in extracellular recordings such as electroencephalograms (EEG), electrocorticograms (ECoG), or local field potential recordings (LFP) is a long-standing method in neurophysiology. In this context, ‘noise’ refers to the variation in the integrated potentials recorded extracellularly from active neuronal assemblies. At the single-cell level, analyzing subthreshold voltage noise allows us to describe events at the cell membrane, such as the stochastic opening and closing of membrane channels and random synaptic currents [34, 35, 76]. These events are crucial to neuronal functions such as signal integration, gain modulation, plasticity, excitability, timing, and synchronization within networks [77–79]. Several studies have shown the role of subthreshold oscillations in olfactory processing [33, 80–82]. Following established EEG recording methods, we analyzed mitral cell membrane potential noise via Fourier transformation to decompose oscillation frequencies and quantify the average power across frequency bands and spectral power density (PSD). A comparison of the current PSD from mitral cells in healthy brains with mitral cells in inflamed brains showed an upward shift of the PSD curve and an increase of average power in the diseased tissue, particularly at lower frequencies (e.g., delta band 0.1–3 Hz) where the magnitude of power is dominated by the activity of membrane channels such as Na^+^ and K^+^ channels [35, 76]. In our experiments, the impact of the Na^+^ conductance on the current fluctuations is negligible due to the depolarized holding potential of −30 mV, which results in the inactivation of Na^+^ channels, thus making a modification of K^+^ channels the likely underlying change in PSD during EAE. This is confirmed by our recordings using a K^+^-free Cs^+^-based pipette solution, which reduced the K^+^ conductance and thereby shifted the PSD to lower power. Interestingly, alterations in PSD power at lower frequencies have also been observed in classical “macroscopic” signals such as EEG in the LPS-induced neuroinflammation model [83].

There are some limitations to our study. The mice suffering from EAE exhibited significant differences in the two olfactory behavioral tests compared to their healthy control group. We controlled for group differences due to stress response by analyzing additional behaviors, such as the overall sum of sniffing time and self-grooming behavior. However, in our study, we cannot exclude the possibility that anxiety may be altered in EAE, as has been previously reported [84, 85].

Even when the disruption of mitral cell function alone can account for olfactory dysfunction, it is reasonable to assume that mitral cells are not the sole neuron type whose homeostasis is disrupted in an inflammatory environment, and that multiple components of the processing circuit are affected. This is also exhibited by the loss of TH-positive interneurons and the immigration of immature DCX-positive neurons, which we detected by immunohistochemical staining at the peak of EAE in the olfactory bulb.

TH-positive juxtaglomerular cells express glutamic acid decarboxylase (GAD), the rate-limiting enzyme for GABA biosynthesis, and co-release dopamine and GABA [86]. TH-positive cells establish synaptic contacts with the afferent olfactory receptor neuron terminals and/or with external tufted cells and form extensive interglomerular connections [87, 88], participating in the early steps of odor information processing that occur in the input layer of the OB. However, increasing evidence points to TH cells as a susceptible cell type. Using a paradigm of naris occlusion in adult mice, it has been shown that among different neurochemical types of juxtaglomerular cells, TH-positive cells were the only ones to show increased apoptosis [89]. We have shown, in part, a co-expression of TH and DCX, indicating that TH cells are part of adult-born immature cells. The perturbation of neurogenesis during neuroinflammation has already been reported in the hippocampus and subventricular zone in a cuprizone model of progressive MS [90].

Limited statements can be made regarding the direct impact of microglial activation on olfactory dysfunction and neurodegeneration. The contribution of microglia biology in MS in complex. On one hand, a direct disease-promoting role is discussed, characterized by the production of neurotoxins. On the other hand, a protective role for microglia is also possible; however, their prolonged activity, coupled with the expression of injurious molecules, can drive the neuropathology of MS. The regulation of iron homeostasis in the brain may be an important source of microglia-induced neurotoxicity in MS [91, 92]. Here, we were able to detect microglial activation in the olfactory bulb during EAE, which may serve as an indication of their involvement in neurodegeneration and dysfunction.

Lastly, there may be mechanisms in regions beyond the main olfactory bulb that could lead to olfactory dysfunction. One important mechanism may be the perturbation of the olfactory epithelium in the naris, leading to loss of sensory neurons, which in turn impacts the entire olfactory network [93].

In summary, in the MS model EAE the main olfactory bulb is affected by T cell-dominated immune cell infiltration and microglial activation, leading to neurodegeneration in vulnerable neuronal cell types. Perturbation of ion channel expression, primarily potassium channel function, was present in the susceptible mitral cells. These changes give a viable explanation of the olfactory dysfunction already seen in the early phases of the disease.

## Materials and methods

### Animal use approval

Female C57BL/6 J mice from Charles River were kept under specific pathogen-free conditions in the central animal facility of the University Medical Center Hamburg-Eppendorf and used for experiments at the age of 9–11 weeks. Mice were housed in a facility at 24 ± 2 °C with 55–65% humidity with a 12 h light/dark cycle and had free access to food and water. For behavioral experiments, animals were housed in an inverted light/dark cycle to enable the performance of the animal experiments in the animal’s active day phase. Animals were adapted to the inverted light/dark cycle at least three weeks in advance. Experiments were carried out under red light and light exposure of less than 25 lx light exposure. The experimental procedures were approved by the local ethics committee (*Behörde für Justiz und Verbraucherschutz* in Hamburg N122/17 and N20/21) in accordance with international and national animal welfare guidelines. We conducted all procedures in accordance with the ARRIVE guidelines [94].

### Experimental autoimmune encephalomyelitis

For the induction of EAE 9–11-week-old female C57BL/6 J mice were anesthetized with isoflurane 1% to 2% v/v oxygen and immunized subcutaneously with 200 µg myelin oligodendrocyte glycoprotein 35–55 (MOG35–55) peptide (peptides and elephants) in emulsion with complete Freund’s adjuvants (BD Difco) containing 4 mg ml^–1^ *Mycobacterium tuberculosis* (BD Difco). 200 ng pertussis toxin (Merck Millipore) was injected intraperitoneally in 100 µl PBS on the day of immunization and two days after. Weight and clinical signs of disease were scored daily from day seven until day 30. Mice were scored for clinical signs by the following system: 0: no clinical deficits; 1: tail weakness; 2: hind limb paresis; 3: partial hind limb paralysis; 3.5: full hind limb paralysis; 4: full hind and forelimb paresis. Animals that reached a score of 4, or 3.5 for more than 7 days, or lost weight of ≥ 25% of starting weight were euthanized according to the regulations of the local animal welfare guidelines. All EAE experiments were performed with an age-matched control group of female C57BL/6 J mice.

### Olfactory detection test

In this behavioral test, mice were assessed for their ability to sequentially detect a control and two novel odors (vanilla and almond, Dr Oetker) during a two-sided olfactory exposure in a test cage. Mice underwent three trials of 4 min each, with one trial involving an empty tube (control) and the other trials involving Whatman paper (0.6 cm Ø) filled tubes containing 5 µl of the test odor (vanilla or almond). Control and test tubes were exchanged simultaneously on both sides of the cages to minimize handling bias. Time spent sniffing in the tube was measured using a video tracking system (Ethovision) and manually by an observer who was blinded to the genotype of the tested animal. Mice were habituated to the environment once daily for five days prior to testing. Analyses were done under red light (< 20 lx). Mice were tested in a healthy condition or during the early phase of EAE (day 9–11 p.i.; severity score ≤ 1.5) and in the chronic phase of EAE (day 28 p.i.). The experimental materials were cleaned with soap, water, 70% ethanol before and after each contact with an animal.

### Olfactory detection test with 2,5-dihydro-2,4,5-trimethylthiazoline (TMT)

For the olfactory detection test a Whatman paper of 1 × 2 cm nourished with 10 µl of 15% TMT was fixed to the side of a test cage (325 × 170 × 140 mm). Mice were placed in the test cage immediately. The cages were closed to avoid diffusion of the odor and exposition to other environmental stimuli. Reaction to the rising concentration of the odor was measured by time of immobility in a trial of 12 by the video tracking system EthoVision (Noldus) [95] and manually by an observer, blinded to the treatment of the tested animal. The experimenter trained himself until an agreement of at least 90% consistency between two analyses performed at different times on the same mouse, as calculated with the Reliability Test provided by The Observer (Noldus) (having 1 s as the maximal time discrepancy between two evaluations). Analyses were done under red light < 20 lx. Mice were tested in healthy condition or at the early phase of EAE (day 10–12 p.i.; severity score ≤ 1.5) and chronic phase of EAE (day 28 p.i.). The experimental material was cleaned with soap, water, and ethanol (70%) before and after each contact with an animal.

### Mouse tissue preparation for immunohistochemistry

For immunohistochemistry/structural neuronal analyses mice were deeply anesthetized intraperitoneally with 100 ml of solution (10 mg ml^–1^ of esketamine hydrochloride (Pfizer), 1.6 mg ml^–1^ of xylazine hydrochloride (Bayer) dissolved in water) per 10 g of body weight. The cardial perfusion with 1% PBS solution were followed by injection of a 4% paraformaldehyde solution.

### Histopathology

For histopathology of EAE mice, brain tissue was fixed with 4% paraformaldehyde as mentioned above. After four hours of fixation tissue was stored in PBS, decalcifies, dehydrated, cast in paraffin and stained according to the standard procedures of the UKE Mouse Pathology Facility as previously described [96]. For orientation in the tissue, hematoxylin staining (blue color) was done, followed by anti-CD3 primary antibodies (rabbit IgG, Abcam Cat# ab16669, RRID: AB_443425), CD45 (rat IgG, Novus; Cat# NB110-63609, AB_1236795) and anti-Iba1 (rabbit IgG, Fujifilm Wako Shibayagi; Cat# 019–19741, RRID: AB_839504) that were visualized using the avidin–biotin complex technique with 3,3’-diaminobenzidine (brown stain). Slides were imaged with a NanoZoomer 2.0-RS digital slide scanner. CD3^+^, CD45^+^, Iba1^+^, P2Y12^+^, TMEM119^+^ and GFAP^+^ cells were analyzed using the NDP.view2 software (Hamamatsu) and the open-source software Qpath (https://qupath.github.io) using the semi-automated positive cell detection tool. Three slices of the olfactory bulb per animal were analyzed. The mean of all technical replicates was measured to obtain the result of one biological replicate.

### Immunofluorescence

After storage in 4% paraformaldehyde for four hours after preparation brains were transferred to 30% sucrose at 4 °C. Embedding of the tissue was done with cryoprotective medium (Tissue Tek® from Sakura) before cryosectioning of 12 µm coronal slices of the olfactory bulb done with a freezing microtome (Leica Jung CM3000). Cryosections of olfactory bulb were incubated in 10% of normal donkey serum containing 0.1% of Triton X-100 and were subsequently stained with antibodies against the following antigens: Calbindin (chicken, Synaptic Systems; Cat# 214,006, RRID: AB_2619903), Calretinin (chicken, Synaptic Systems; Cat# 214,106, RRID: AB_2619909), Doublecortin (rabbit, Abcam,; Cat# 18,723, RRID: AB_732011), HuC/D (mouse, Molecular Probes; Cat# A-21271, RRID: AB_221448), NeuN (chicken, Millipore; Cat# ABN91, RRID: AB_11205760), Reelin (mouse, Millipore; Cat# MAB5364, RRID: AB_2179313), PSD95 (mouse, Abcam; Cat# ab2723, RRID: AB_303248), SMI31 (mouse, BioLegend; Cat# 801,601, RRID: AB_2715851), SMI32 (mouse, BioLegend; Cat# 801,701, RRID: AB_2715852), Synapsin 1 (guinea pig, Synaptic Systems; Cat# 106 011, RRID:AB_2619772), and Tyrosine Hydroxylase (mouse, Synaptic Systems; Cat# 213104, RRID:AB_2619897), As secondary antibodies, we used Alexa Fluor 488–coupled donkey antibodies recognizing chicken IgG (1:600, Jackson Immunoresearch; Cat# 703–545-155, RRID: AB_2340375), rabbit IgG (1:600, Jackson Immunoresearch; Cat# 711–546-152, RRID: AB_2340619) and mouse IgG (1:600, Abcam; Cat# ab150105, RRID: AB_2732856), Alexa Fluor 647–coupled donkey antibodies recognizing rat IgG(1:600, Abcam; Abcam; Cat# ab150159, RRID: AB_2566823) and rabbit IgG (1:600, Abcam; Cat# ab150075, RRID: AB_2752244). To avoid background labeling when using anti-mouse primary antibodies in inflamed mouse tissue, we used a Fab fragment anti-mouse IgG (1:200, Jackson Immunoresearch, Cat# 715–007- 003, RRID: AB_2307338) in 0.1% of Triton X-100 for one hour before using the anti-mouse antibody. Images were acquired using a Zeiss LSM 700 confocal microscope. Samples were analyzed by the open-source package Fiji based on ImageJ. Reelin-positive, HuC/D-positive, DCX-positive and TH-positive cells were counted manually within their respective layer. Mitral cells were calculated per mm of the MCL, while tufted cells were quantified per mm of the GL. To quantify these, the length of the GL was measured at its lower edge, as some tufted cells are localized not only within the GL but also adjacent to it. All other cell types were quantified per the area indicated on the y-axis of the figures. The quantification of Calbindin-positive, Calretinin-positive and NeuN-positive cells was automatized after binarization of the selected area using the threshold *triangle*. For staining of neurofilaments in the IPL of olfactory bulb slices we used Sternberger® purified anti-neurofilament H (NF-H) monoclonal mouse SMI31 and SMI32 antibodies to detect phosphorylated and non-phosphorylated neurofilament H, respectively. To avoid cross-reaction of the secondary antibody we combined direct and indirect immunofluorescence. For that, we coupled the SMI31 primary antibody directly to Alexa Fluor 488 dye using the APEX^®^ labeling kit (Invitrogen^®^, fisher scientific) according to manufacturer instructions. After blocking the slices for 1 h at room temperature with 0.5% normal goat serum, 0.05% Triton-X-100 in phosphate buffered saline we performed indirect staining with SMI32 antibody and goat anti-mouse IgG Alexa Fluor 555 (Invitrogen^®^, fisher scientific). After washing out the secondary antibody we affiliated the direct staining with the coupled primary antibody SMI31-488. Additionally, DAPI (5 µM) was used as a nuclei marker. All brain slices of the healthy control cohort and of EAE animals were treated in parallel using an antibody mastermix. Images were acquired alternating between both groups using a Nikon eC1 confocal microscope in frame lambda mode with the same settings for laser power and gain. Analysis of mean fluorescence intensity in the IPL was performed by Nikon EZ-C1 viewer and Fiji software using maximum intensity 2D projections of 5 µm z-stacks (10 images) after subtraction of background and thresholding images with the isodata algorithm. Three slices of the olfactory bulb per animal were analyzed by immunofluorescence. The mean of all technical replicates was measured to obtain the result of one biological replicate.

### Isolation of CNS-infiltrating immune cells and flow cytometry

For the characterization of the CNS-infiltrating immune cells from different phases of EAE (onset and acute EAE), animals were shortly perfused with ice-cold 1 × PBS to remove blood from the intracranial vessels, the olfactory bulb and spinal cord were isolated and the tissue minced with a scalpel, and incubated with agitation in RPMI 1640 (PAA) containing collagenase A (1 mg ml^−1^) (Roche) and recombinant ribonuclease-free deoxyribonuclease I (0.1 mg ml^−1^) (Roche) for 45 min at 37 °C. Tissue was triturated through a 100 μm cell strainer and washed with PBS (pellet was centrifuged at 300 ×*g* for 10 min at 4 °C). The homogenized spinal cord tissue was resuspended in 30% isotonic Percoll (GE Healthcare) and carefully underlaid with 78% isotonic Percoll. After gradient centrifugation (1500 ×*g* for 30 min at 4 °C), CNS-infiltrating immune cells were recovered from the gradient interphase and washed in ice-cold PBS. Due to limited sample material the gradient centrifugation step was omitted for olfactory tissue. The antibodies and the respective antigen, host species, supplier, catalog number, clone, and dilution are listed in Table S1. Dead cells were excluded using Pacific Orange NHS (Invitrogen, #P30253). 123count eBeads (Invitrogen) were used to determine absolute cell counts. Data were acquired on an LSR II FACS analyzer (BD Biosciences). Data analysis was performed with the FlowJo v.10 analysis software (FlowJo LLC).

### Electrophysiological recordings

Electrophysiological recordings were performed in acute brain slices of healthy wildtype mice and age and sex-matched mice at the acute phase of the EAE (day 13–16 p.i.). Dissection was performed as previously described [65]. Briefly, olfactory bulbs were quickly transferred into a chilled slicing artificial cerebrospinal fluid (ACSF) that contained (in mM): NaCl, 83; NaHCO_3_, 26.2; NaH_2_PO_4_, 1; KCl, 2.5; sucrose, 70; D-glucose, 20; CaCl2, 0.5; MgSO_4_, 2.5. ACSF was continuously gassed with carbogen (95% O_2_–5% CO_2_). 220-µm horizontal olfactory bulb slices were cut using a vibratome (Leica VT1200S). Brain slices were stored in ACSF containing (in mM): NaCl, 120; NaHCO_3_, 26; NaH_2_PO_4_, 1; KCl, 2.5; D-glucose, 2.8; CaCl_2_, 2; MgCl_2_, 1. Storage lasted for 30 min at 30 °C and at least 15 min at room temperature before starting experiments. Throughout the experiments, brain slices were superfused with ACSF. Mitral cells of the main olfactory bulb were identified for patch clamp recordings by their location, size and shape. Whole-cell configuration was employed using patch pipettes with a resistance of 3–5 MOhm. Recordings were performed using MultiClamp 700B amplifier and pCLAMP 10 software (Molecular Devices), digitized (Digidata 1440A, Molecular Devices) at 10–20 kHz and filtered (Bessel filter, 2 kHz). The standard pipette solution contained (in mM): NaCl, 10; potassium gluconate, 105; K3-citrate, 20; Hepes, 10; MgCl_2_, 0.5; Mg-ATP, 5; Na-GTP, 0.5. To examine the current–voltage relationship cells were held in voltage clamp at −50 mV and voltage steps from −100 to 20 mV (duration 500 ms, delta 15 mV) were applied. To determine I_h_ current in voltage clamp we applied hyperpolarizing voltage steps from −60 to −120 mV and quantified the amplitude of I_h_ as the difference between inward current at the beginning and at the end of the voltage step. To investigate the halothane-induced current 3 mM halothane in ACSF was obtained by dilution of halothane-saturated ACSF as described previously for Ringer solution [97] and applied for 25 min with the perfusion system driven by a peristaltic pump (Reglo, Ismatec) at a flow rate of 2 ml min^–1^. Cells were held at −30 mV to increase the driving force for potassium currents and short voltage steps of −5 mV were applied every 20 s during the recording to monitor the membrane conductance. To compare the current noise (PSD) of mitral cells from EAE animals and healthy controls 20 s of current traces recorded at a holding potential of –30 mV before halothane application were used. For examining the effect of K^+^ channel on current noise we used 7.5 s traces of mitral cells recorded with a standard intracellular solution or with Cs-based intracellular solution (in mM: CsMeSO_3_, 120; TEA-Cl, 15; 4-AP, 5; NaCl, 10; MgCl_2_, 1; EGTA, 1.1; Mg-ATP, 3; Na-GTP, 0.5; HEPES, 10) at a holding potential of −30 mV in the presence of TTX (0.5 µM), NBQX (10 µM), D-APV (50 µM) and gabazine (10 µM). Sampling frequency was 10 kHz for all noise analysis data sets. Series resistance was monitored closely during all recordings and cells exceeding 20 MOhm or showing a change of more than 20% were excluded from analysis. All voltage data are not corrected for liquid junction potential (LJP −18 mV). Data were analyzed using Clampfit10 (Molecular Devices), Excel (Microsoft Office) and Origin 2021 b (OriginLab) software. For spectral composition and PSD analysis the decimation in time FFT (Fast Fourier Transform) algorithm in Clampfit10 was used without windowing and segment overlap. For quantification of spectral power, we averaged the integrals of the linear power spectra in the following frequency bands: delta (0.1–3 Hz), theta (3–8 Hz), alpha (8–12 Hz), beta (12–30 Hz) and gamma (30-100 Hz).

### Nucleus isolation

Mice were sacrificed with CO_2_ and perfused with ice-cold PBS. Olfactory bulb tissue was harvested and snap-frozen in –80 °C until further analysis. For nuclei isolation olfactory bulb tissues were homogenized in EZ lysis buffer (Sigma Aldrich, NUC101) with a glass douncer (Sigma-Aldrich, D9063). After 3 min incubation on ice cell suspension was then centrifuged by 500 × g for 4 min at 4 °C. Nuclei were washed once with the EZ lysis buffer and twice in the nucleus incubation buffer (MgCl_2_ 2 mM, KCl 25 mM, Glycerophosphate 65 mM, Sucrose 340 mM, glycerol 5%, ethylenediaminetetraacetic acid 1 mM, bovine serum albumin (BSA) 1%) and filtered through a 30 µm pre-separation filter (Miltenyi Biotech, 130-041-407).

### Fluorescence-activated cell sorting

The nuclei suspensions were stained in nucleus incubation buffer for ten minutes with anti-HuC/D labeled to Alexa647 (1:300, rabbit, Abcam; Cat# ab237235, RRID: AB_2864321) and propidium iodide (PI) (1:1000, Thermo Fisher Scientific, Cat# P1304MP). PI-positive HuC/D positive nuclei were sorted in PBS containing 0.4% BSA using a BD Aria III cell sorter (BD Biosciences). For validation of upregulated genes in the snRNA-seq dataset, PI-positive HuC/D highly positive nuclei, defined as the 2% of PI-positive nuclei expressing HuC/D the highest, were sorted in 0.4% BSA and samples were immediately processed for real time PCR.

### Real-time PCR

The RNA of the sorted samples was extracted using the RNeasy Mini Kit (Qiagen) and subsequently reverse-transcribed to complementary DNA with the RevertAid H Minus First Strand cDNA Synthesis Kit (Thermo Fisher Scientific) according to the manufacturer’s instructions. The analysis of the gene expression by real-time PCR was performed in the ABI Prism 7900 HT Fast Real-Time PCR System (Applied Biosystems) using TaqMan Gene Expression Assays (Thermo Fisher Scientific) of *Gbp6* (Mm00843395_m1), *Lef1* (Mm00550265_m1), *Kcnk5* (Mm00498900_m1), *Tthy2* (Mm00499743_m1) and *Tbp* (Mm01277042_m1). Gene expression was calculated as 2^–Δ*Ct*^ relative to *Tbp* as the endogenous control.

### Single-cell RNA sequencing

mRNA library preparation was performed according to the standard 10X Genomics protocol with the Chromium Next GEM Single Cell 3’GEM, Library & Gel Bead Kit v3.1 (Cat# 1,000,075, 10 × Genomics). Briefly, gel bead emulsion (GEMs) was generated containing single nuclei and a master mix with reagents in the Chromium Controller followed by cell lysis and reverse transcription of RNA, cDNA amplification, enzymatic fragmentation, 5´adaptor attachment and sample indexing. On average around 25,000 nuclei were loaded on each channel of the Chromium instrument. To minimize batch effects, EAE and control samples were distributed evenly in the Chromium controller. Libraries were pooled and sequenced on an Illumina NovaSeq6000 platform (paired end sequencing, 2 × 100 bp read length, 500 million reads per sample).

### Data processing

The Cell Ranger Software (version 5, 10X Genomics) was used for demultiplexing and alignment of sequencing reads to the mouse transcriptome. SnRNA-seq data were processed using the Seurat package (version 4.4.0, Satija Lab). Quality control metrics, including the percentage of mitochondrial gene expression (percent.mt), were calculated using *PercentageFeatureSet* with a pattern set to ‘‘^mt-’’ to identify mitochondrial genes. Samples were filtered to exclude cells with an abnormal number of features or mitochondrial content, as these may indicate dead or dying cells. Data normalization was performed using the *NormalizeData* method with default parameters to mitigate the effects of technical variability. Variable features were identified using the *FindVariableFeatures* function, employing the vst method, with the number of features set to 2000, to capture the most informative genes for downstream analysis. To minimize batch effects and integrate data from different samples, we used the *SelectIntegrationFeatures* function to identify common features across all samples. Integration anchors were computed with *FindIntegrationAnchors*, and the datasets were integrated using *IntegrateData*. This integrated dataset was then scaled (*ScaleData*) and subjected to principal component analysis (PCA) using *RunPCA* with the number of principal components set to 30. UMAP (Uniform Manifold Approximation and Projection) was employed for dimensionality reduction (*RunUMAP*), using the first 20 PCA dimensions. Cells were clustered using the FindNeighbors (considering the first 20 PCA dimensions) and *FindClusters* functions, with the resolution parameter set to 0.1 to fine-tune the granularity of clustering. Differential expression analysis was conducted using *FindAllMarkers* with parameters set to identify only positive markers with a minimum percentage of cells expressing the gene (*min.pct*) at 0.25 and a log fold change threshold (*logfc.threshold*) of 4. Top markers were selected for further visualization in a heatmap (*DoHeatmap*) and violin plots (VlnPlot) to explore expression patterns across clusters. For selected clusters, sub-clustering was performed to further resolve cellular heterogeneity. This involved repeating the normalization, PCA, UMAP, and clustering steps on the subset data. Differential expression analysis was then conducted within these sub-clusters to identify cluster-specific marker genes. Functional enrichment analysis was executed using the *clusterProfiler* package and the *org.Mm.eg.db* database for mouse gene annotations. Enriched Gene Ontology (GO) terms in the Molecular Function category were identified for significant genes in each cluster, adjusting for multiple comparisons using the Benjamini–Hochberg method *(pAdjustMethod* = ’’BH’’) with a threshold (*qvalueCutoff*) of 0.05.

### Software and packages

Data analysis was performed in R (version 4.3.2) using packages Seurat (for snRNA-seq data analysis), ggplot2 and ggsci (for data visualization), dplyr (for data manipulation), clusterProfiler, enrichplot, and org.Mm.eg.db (for functional enrichment analysis). Specific parameters and thresholds were chosen based on standard practices in the field and were adjusted as necessary to accommodate the unique characteristics of the dataset.

### Statistical analysis

Graph Pad Prism (version 8) and R software (version 4,3,2 64-bit) were used general statistical analyses (such as mean, SEM, t-tests, Mann–Whitney-U-test, two-way ANOVA, Kolmogorov–Smirnov-Test and Tukey’s multiple comparisons post hoc test. All tests were two-tailed and level of significance was set at *P* < 0.05. For consistency of comparisons, significance in all figures is denoted as follows: *P < 0.05, **P < 0.01, *P < 0.001. No statistical test was used to pre-determine sample size. No samples were excluded. All attempts at replication were successful. The nature and number of samples analyzed (defined as *n*) in each experiment are listed in the figure legends. The number of independent experiments is also listed. The investigators were blinded when quantifying immunofluorescence results. For snRNA-seq experiments, the statistical treatments are described in those sections.

## Supplementary Information

Supplementary material 1.

## Data Availability

Data generated for this study are available through the Gene Expression Omnibus under accession number GSE268675.
