# Supplementary material for: Neuroinflammation causes mitral cell dysfunction and olfactory impairment in a multiple sclerosis model
Source: J Neuroinflammation. 2025 Mar 8;22:71. doi: 10.1186/s12974-025-03388-5 (PMC11889885; doi:10.1186/s12974-025-03388-5)
Supplement: Supplementary file 1 — Supplementary material 1. [file 12974_2025_3388_MOESM1_ESM.pdf]

SUPPLEMENTAL MATERIAL

Neuroinflammation causes mitral cell dysfunction and olfactory impairment in a multiple sclerosis model

Charlotte Schubert[1], Kristina Schulz[2], Jana K. Sonner[1], Alexandros Hadjilaou[1,3], Anna-Lena Seemann[1,2], Janine Gierke[2], Vanessa Vieira[1], Nina Meurs[1], Marcel S. Woo[1], Christian Lohr[2], Fabio Morellini[4], Daniela Hirnet[2,5], Manuel A. Friese[1,5,*]

[1]Institute of Neuroimmunology and Multiple Sclerosis (INIMS), University Medical Center Hamburg-Eppendorf, Hamburg, Germany

[2]Institute of Cell and Systems Biology of Animals, University of Hamburg, Hamburg, Germany

[3]Protozoa Immunology, Bernhard Nocht Institute for Tropical Medicine, Hamburg, Germany.

[4]Research Group Behavioral Biology, Center for Molecular Neurobiology (ZMNH), University Medical Center Hamburg-Eppendorf, Hamburg, Germany

[5]These authors contributed equally

*Correspondence: manuel.friese@zmnh.uni-hamburg.de

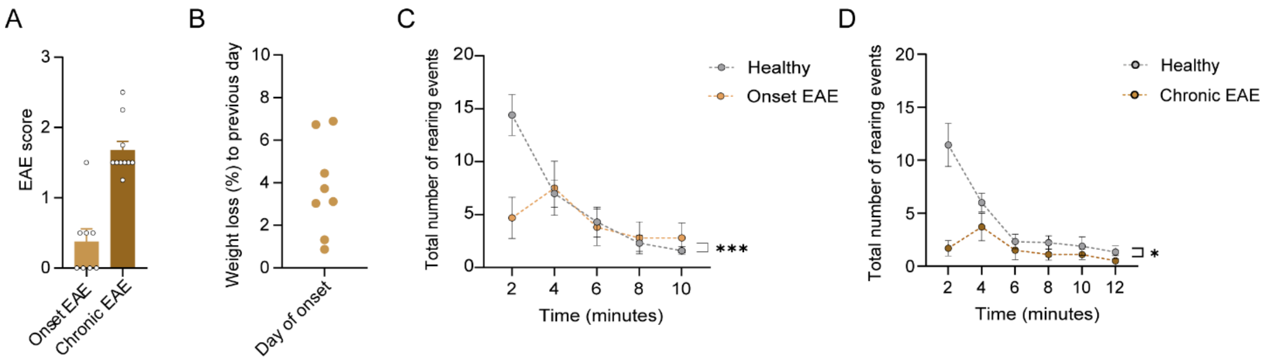

Fig. S1 EAE leads to impairment in olfaction during EAE.

(**A**) EAE score at time of behavioral testing at onset of EAE (d9 p.i., *n* = 8) and chronic EAE (d25 p.i., *n* = 10).
(**B**) Weight loss at the day of behavioral testing at onset of EAE in percent to the previous day (*n* = 8).
(**C**) Number of rearing events in healthy mice and EAE mice at disease onset (EAE score ≤1.5 and detectable weight loss. *n* = 10 per group).
(**D**) Number of rearing events during TMT-based olfactory detection test analyzed in bins of 2 minutes in healthy mice (*n* = 9) and in EAE mice at chronic stage of EAE (d25 p.i.. *n* = 10). Statistics were performed by REML mixed-effects model with Šídák multiple comparison; *$P < 0.05$. ***$P < 0.001$.

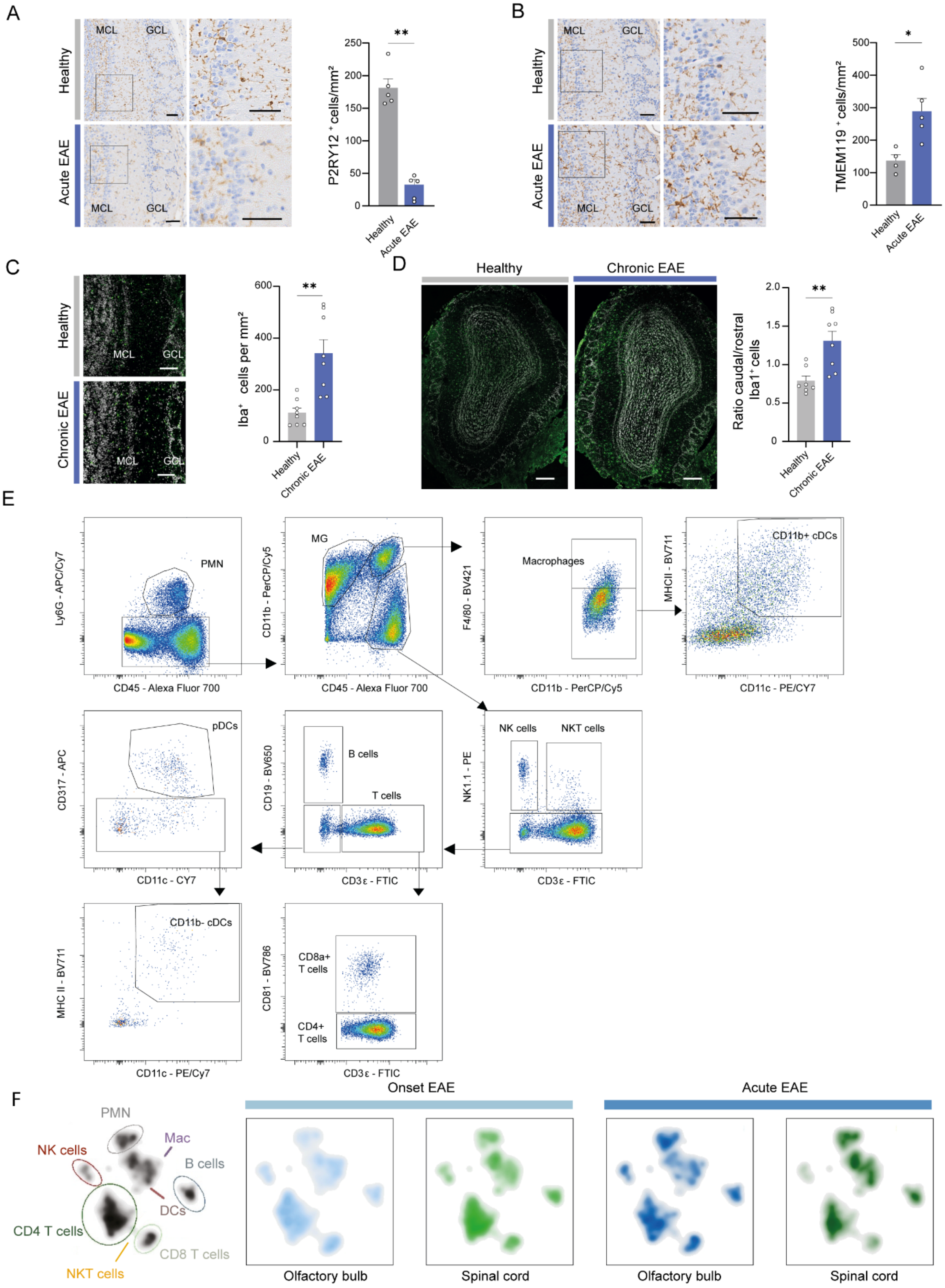
A
Healthy
Acute EAE
MCL
GCL
P2RY12 + cells/mm²
**
B
TMEM119 + cells/mm²
*
C
Chronic EAE
Iba+ cells per mm²
D
Ratio caudal/rostral Iba1+ cells
E
PMN
Ly6G - APC/Cy7
CD45 - Alexa Fluor 700
MG
CD11b - PerCP/Cy5
Macrophages
F4/80 - BV421
CD11b - PerCP/Cy5
CD11b+ cDCs
MHCII - BV711
CD11c - PE/CY7
pDCs
CD317 - APC
CD11c - CY7
B cells
T cells
CD19 - BV650
CD3ε - FTIC
NK cells
NKT cells
NK1.1 - PE
CD11b- cDCs
MHC II - BV711
CD11c - PE/Cy7
CD8a+ T cells
CD4+ T cells
CD81 - BV786
F
PMN
NK cells
Mac
B cells
DCs
CD4 T cells
CD8 T cells
NKT cells
Onset EAE
Acute EAE
Olfactory bulb
Spinal cord

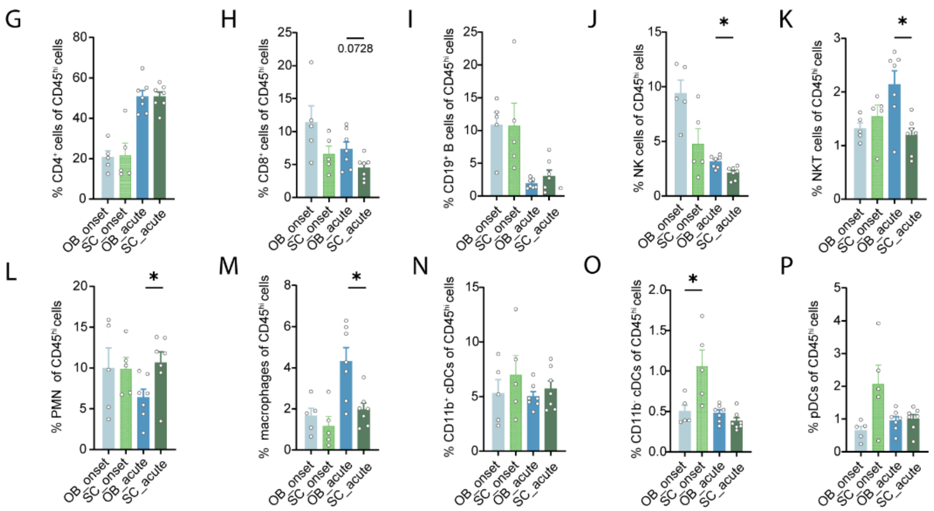

Fig. S2 Immune cell infiltration and glial cell activation in the olfactory bulb during EAE.

(**A**) Representative image with zoom-in (enlarged section displayed by frame in overview image) and quantification of per mm² of $P2Y12^{+}$ microglia in the main olfactory bulb (coronar orientation) in healthy mice and at the acute phase of EAE (d15 p.i.) (*n* = 5 per group). Scale bar = 50 µm.
(**B**) Representative image with zoom-in (enlarged section displayed by frame in overview image) and quantification of per mm² of $TMEM119^{+}$ cells in the main olfactory bulb (coronar orientation) in healthy mice and at the acute phase of EAE (d15 p.i.) (*n* = 5 per group). Scale bar = 50 µm.
(**C**) Representative images with zoom-in (enlarged section displayed by frame in overview image) and and quantification of Iba1+ cells per mm² of olfactory bulb in healthy animals (*n* = 5) and chronic EAE (d30 p.i.. *n* = 5). IBA1 signal depicted in green. DAPI staining in grey. Scale bars = 100 µm.
(**D**) Ratio of Iba1+ cells in the caudal half of the olfactory bulb coronar sections in comparison to the rostral part in healthy animals and the chronic stage of EAE (d30 p.i.). IBA1 signal depicted in green. DAPI staining in grey. Scale bars = 200 µm.

(**E**) Gating strategy of analyzed olfactory bulb and spinal cord infiltrating immune cells by flow cytometry. Polymorphonuclear neutrophiles = PMN. MG = Microglia. CD11b+ and CD11b– conventional dendritic cells = cDCs. plasmacytoid dendritic cells = pDC. CD8– T cells are referred to as CD4+ T cells. as they are the vast majority comprising this cluster.

(**F**) UMAP plots of infiltrating immune cells merged. at onset of EAE (d9 p.i.. *n* = 5) and at acute EAE (d14 p.i.. *n* = 7) in the olfactory bulb as well as spinal cord. The UMAP of infiltrating immune cells in the olfactory bulb at acute EAE is also shown in Figure 2.
(**G–P**) Frequency of infiltrating immune cells in relation to CD45 high expressing cells ($CD45^{hi}$) in the olfactory bulb and spinal cord. Shown are CD4+ T cells (E) and CD8+ T cells (F). CD19+ B cells (G). NK cells (H). NKT cells (I). PMN (J). macrophages (K). CD11b+ cDCs (L). and CD11b- cDCs (M) and pDCs (N) at onset of EAE (d9 p.i.) and acute EAE (d14 p.i.). Bars show mean values ± s.e.m. Statistical analysis was performed by Mann-Whitney U-Test; $^{*}P < 0.05$. $^{**}P < 0.01$.

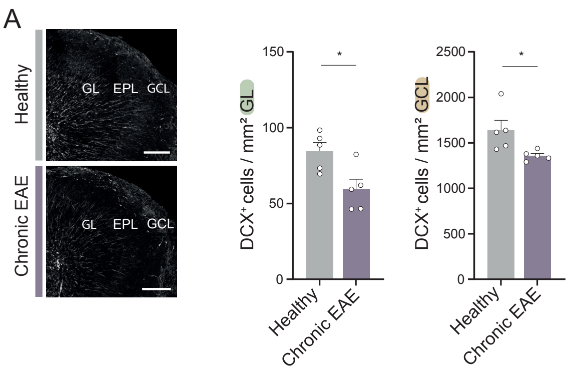

Fig. S3 Structural analyses show cell-type specific neuronal vulnerability during EAE.

(**A**) Representative images and quantification of DCX+ cells per mm² in healthy animals and chronic EAE (d30 p.i.) analyzed in the glomerular layer (GL) and granular cell layer (GCL) (*n* = 5 per group). Scale bars = 100µm. Bars show mean values ± s.e.m. Statistical analysis was performed by Mann-Whitney U-Test; $^{*}P < 0.05$.

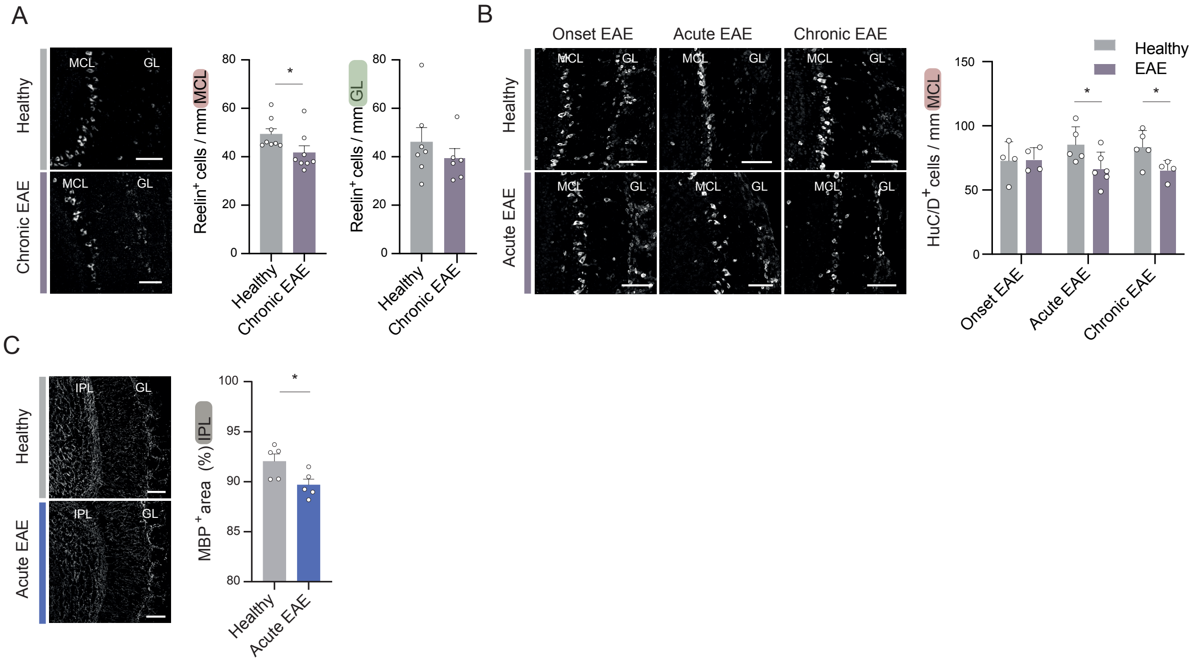

Fig. S4 Mitral cells are specifically susceptible to neuroinflammation in EAE.

(**A**) Representative images and quantification of Reelin⁺ cells per mm mitral cell layer (MCL) and glomerular layer (GL) in chronic EAE mice (d30 p.i.) in comparison to healthy control mice (MC: *n* = 8 per group; GL healthy: *n* = 7*;* GL chronic EAE: *n* = *6*). The healthy control group is identical to that in Fig. 4A. Four healthy animals were sacrificed at the time point equivalent to EAE d15 p.i., and four healthy animals were sacrificed at the time point equivalent to EAE d30 p.i. Scale bars = 100 µm.
(**B**) Representative images and quantification of HuC/D+ cells per mm mitral cell layer (MCL) at onset of EAE (d9 p.i.. *n* = 4). acute stage of EAE (d15 p.i.. *n* = 5) and chronic EAE (d30 p.i.. *n* = 5) and corresponding healthy control animals (*n* = 4. *n* = 5. *n* = 5. respectively) analyzed in the glomerular cell layer (GL) and granular cell layer (GCL). Scale bars = 100 µm.
(**C**) Representative images and quantification of the MBP+ area in the inner plexiform layer (IPL) of the olfactory bulb (*n* = 5 per group). Scale bars = 100 µm.
Bars show mean values ± s.e.m. Statistical analysis was performed by Mann-Whitney U-Test; **P* < 0.05.

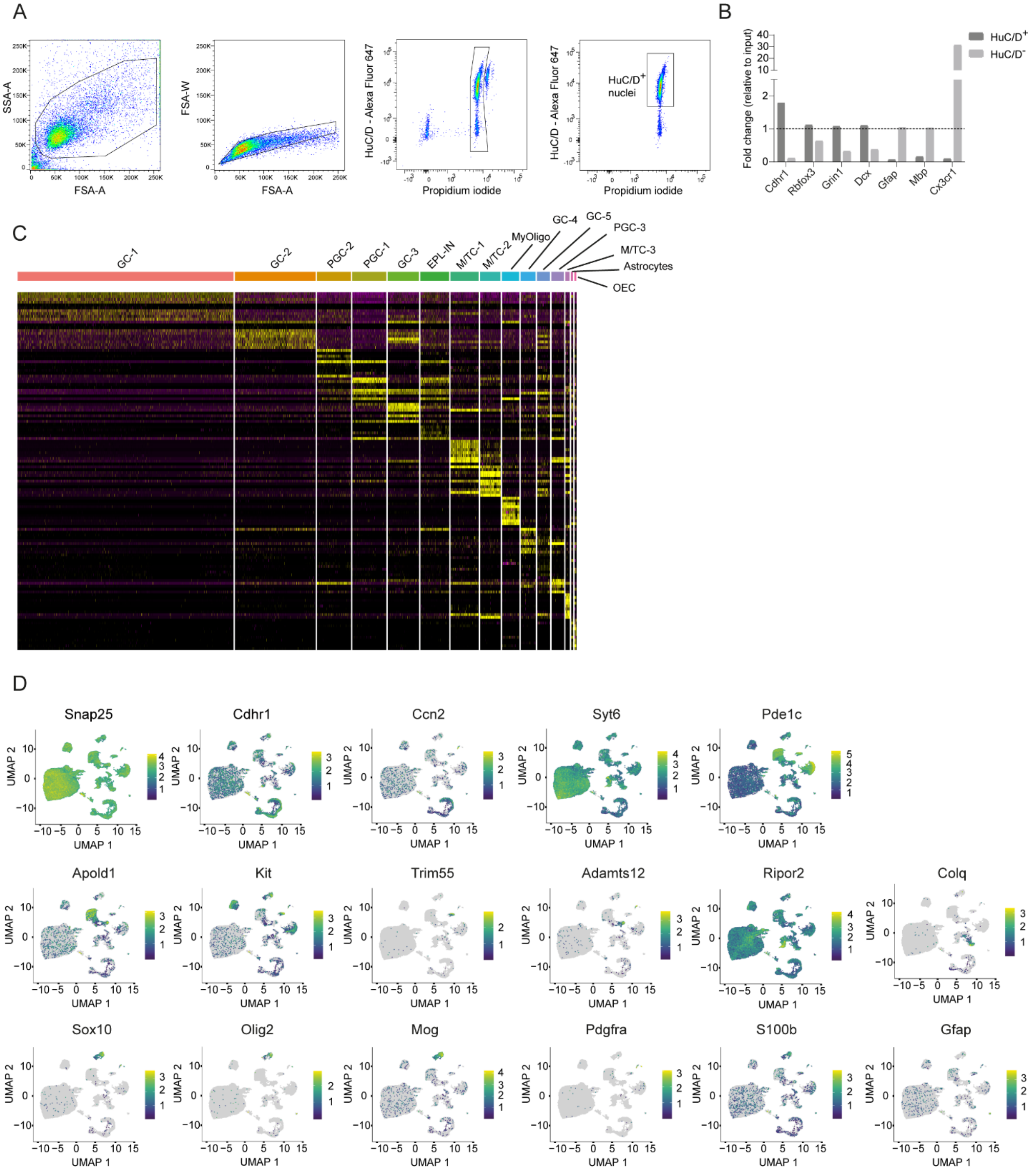

Fig. S5 SnRNA-Seq of the olfactory bulb reveals a heterogeneity of neuronal cell types

(**A**) Gating strategy of cell sorting of HuC/D+ nuclei for snRNA-Seq.
(**B**) Relative mRNA expression of neuronal (*Cdhr1. Rbfox3. Grin1. Dcx*) and non-neuronal (*Gfap. Mbp. Cxcr1*) marker genes of cell sorted HuC/D+ and HuC/D – nuclei (*n* = 1 per group. similar results were obtained in two other validation experiments).
(**C**) Heatmap of marker genes of the 15 cell clusters obtained by snRNA-Seq of HuC/D+ nuclei of the olfactory bulb of healthy animals and at the acute EAE stage (d15 p.i.. *n* = 3 per group).
(**D**) Feature plot of additional genes of interest visualized by UMAP.

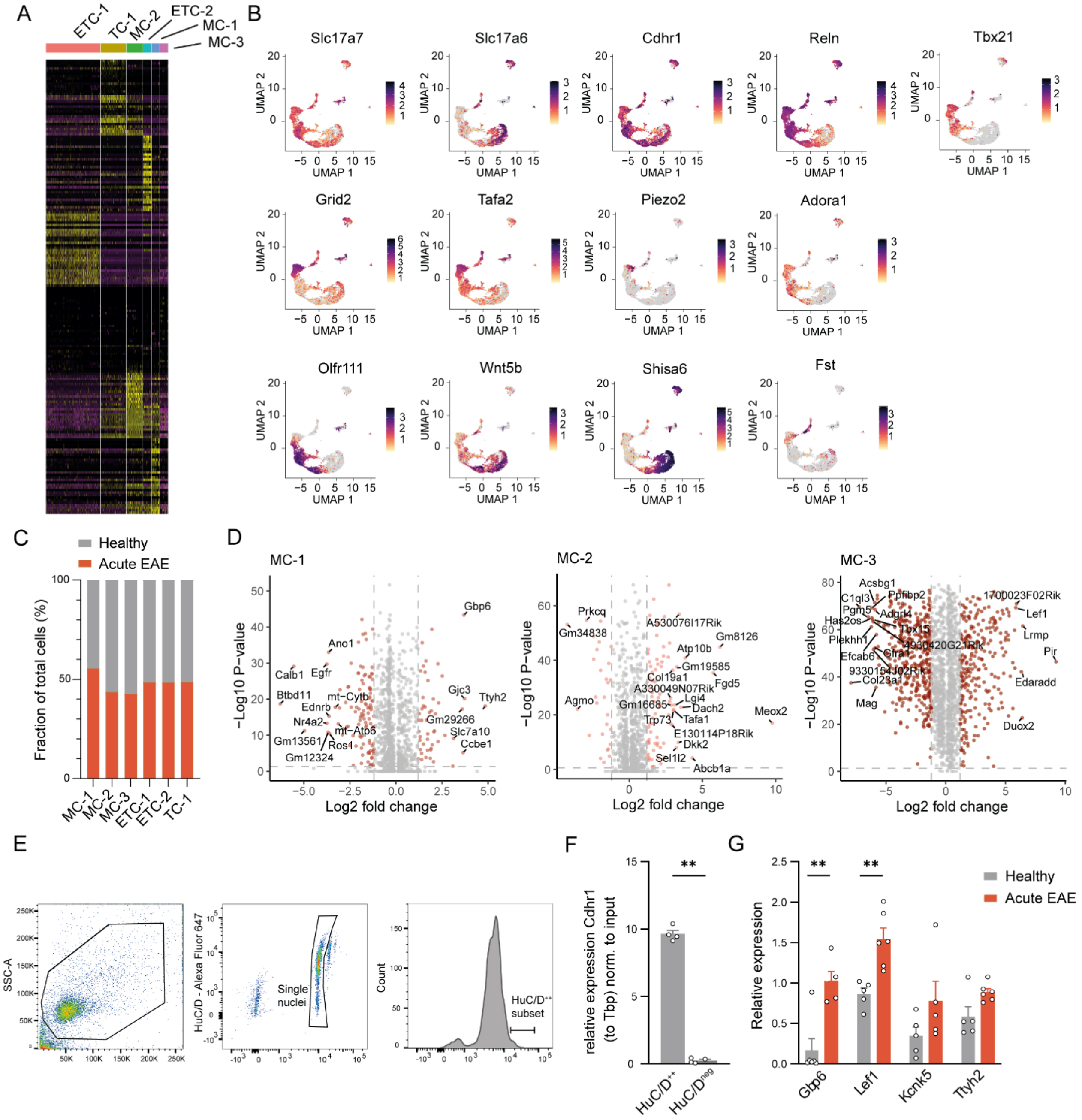

Fig. S6 SnRNA-Seq shows changes in ion channel expression in mitral cells during EAE

(**A**) Heatmap of marker genes of the six subclusters of healthy animals and at the acute EAE stage (d15 p.i.. n = 3 per group).
(**B**) Validation by genes of interest in the six subcluster visualized by UMAP.
(**C**) Fraction of total cells in the olfactory bulb per cluster in healthy mice and acute EAE mice (d15 p.i.) (*n = 3* per group*)*.
(**D**) Differential expressed genes in the three mitral cell clusters (MC-1. MC-2. MC-3) between healthy mice and acute EAE mice (*n* = 3 per group) in snRNA-Seq.
(**F**) Gating strategy of cell sorting of HuC/D++ nuclei for mitral cell enrichment.
(**G**) Relative expression of mitral cell-specific gene *Cdhr1* normalized to the input of olfactory bulb nuclei before cell sorting, measured by quantitative real time PCR.
(**H**) Relative expression of selected genes which where upregulated in the snRNA-seq data set, validated in HuC/D++ sorted nuclei by quantitative real time PCR.

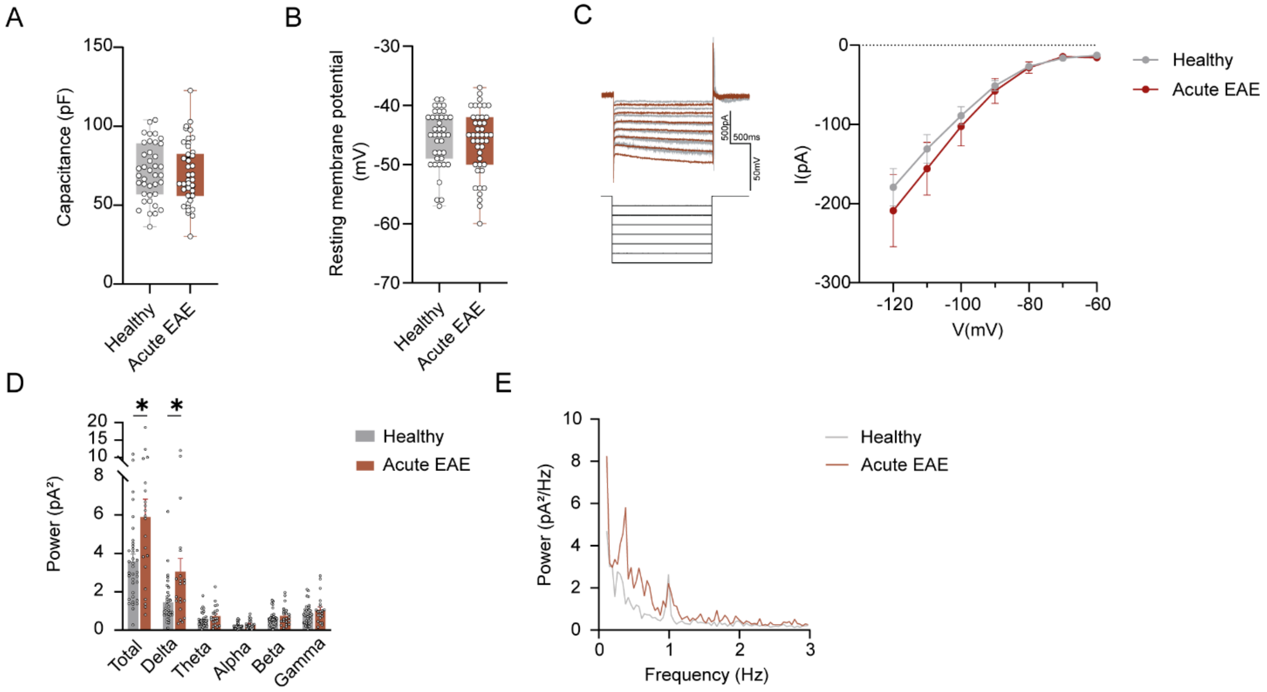

Fig. S7 Intrinsic properties of mitral cells are perturbed during EAE demonstrated in whole clamp analyses.

(**A**) Capacitance acquired by whole clamp analysis of mitral cells in healthy mice (*n* = 39 cells. *n* = 25*)* or acute EAE mice (d13–16 p.i.. *n* = 43 cells. *n* = 20).
(**B**) Resting membrane potential in healthy mice and acute EAE mice (d13–16 p.i.) in mitral cells of the olfactory bulb. Statistical analyses were performed using an unpaired t-test.
(**C**) Voltage step protocol to induce I_h in mitral cells. Representative traces and I/V plot in healthy mice (, *n* = 9cells, *n* = 5 animals) or acute EAE (d13-d16 p.i., *n* = 11 cells, *n* = 5 animals). Difference in evoked I_h inward current quantified as difference between current at beginning and at end of voltage step (-179 +/- 23 pA in healthy mice vs. -221 +/- 45 pA in EAE at V=-120 mV) is not significant (student´s t-test P=0,58).
(**D**) Average power as integral of the corresponding linear spectrograms in the following frequency bands: delta (0.1–3 Hz), theta (3–8 Hz), alpha (8–12 Hz), beta (12–30 Hz) and gamma (30–100 Hz) and in total over all frequencies in healthy (*n* = 38 cells, *n* = 31 animals) vs. acute EAE (d13–16 p.i., *n* = 32 cells, *n* = 12 animals).
(**E**s Averaged linear spectrogram of healthy and EAE mitral cells in delta band (0.1–3 Hz) of the same cohort.

Table S1. List of antibodies used in flow cytometry.

Antigen	Supplier	Prod. no.	RRID	Species	Flow cytometry
CD3ε FITC	BioLegend	100306	AB_312671	Armenian Hamster	1:100
CD8α BV785	BioLegend	100750	AB_2562610	Rat	1:200
CD11b PerCP/Cy5.5	BioLegend	101228	AB_893233	Rat	1:400
CD11c PE/Cy7	BioLegend	117318	AB_493569	Hamster	1:300
CD19 BV650	BioLegend	115541	AB_11204087	Rat	1:400
CD45 Alexa Fluor 700	BioLegend	103128	AB_493715	Rat	1:200
CD317 APC	BioLegend	127016	AB_1967101	Rat	1:100
F4/80 BV421	BioLegend	123137	AB_11203717	Rat	1:100
I-A/I-E BV711	BioLegend	107643	AB_2565976	Rat	1:400
Ly6G APC/Cy7	BioLegend	127623	AB_10640819	Rat	1:400
NK1.1 PE	Thermo Fisher Scientific	12-5941-82	AB_466050	Mouse	1:300

Pacific Orange-NHS (Invitrogen. #P30253) used at 1 μM as viability stain.

Table S2. Quality control of single-nucleus RNA-seq data set.

Cluster	Number of cells	Avg nCount RNA	Avg feature RNA	Avg percent mito
0	29547	4423.07	1762.62	0.007
1	11028	4239.20	1755.72	0.005
2	4657	5659.33	2152.65	0.008
3	4657	2423.02	1219.76	0.011
4	4269	4959.29	1839.11	0.005
5	3941	8757.45	2456.65	0.007
6	3863	11965.38	3101.45	0.013
7	2804	8098.22	2595.75	0.010
8	2377	3855.42	1597.18	0.017
9	2053	4533.45	1805.41	0.011
10	1780	7272.30	2498.09	0.007
11	1729	4477.86	1787.64	0.010
12	585	7531.91	2516.27	0.005
13	279	4627.94	1967.92	0.017
14	266	3436.44	1658.77	0.051
